# Matrix Li–Yau–Hamilton estimates under Ricci flow and parabolic frequency

**DOI:** 10.1007/s00526-024-02668-x

**Published:** 2024-02-26

**Authors:** Xiaolong Li, Qi S. Zhang

**Affiliations:** 1 Department of Mathematics, Statistics and Physics, Wichita State University, Wichita, KS 67260 USA; 2 Department of Mathematics, University of California, Riverside, Riverside, CA 92521 USA

**Keywords:** Primary 53E20, Secondary 58J35, 35K05

## Abstract

We prove matrix Li–Yau–Hamilton estimates for positive solutions to the heat equation and the backward conjugate heat equation, both coupled with the Ricci flow. We then apply these estimates to establish the monotonicity of parabolic frequencies up to correction factors. As applications, we obtain some unique continuation results under the nonnegativity of sectional or complex sectional curvature.

## Introduction

### Matrix Li–Yau–Hamilton estimates

In their seminal paper [49], P. Li and S.-T. Yau developed fundamental gradient estimates for positive solutions to the heat equation on a Riemannian manifold. In particular, they proved that if $u:M^n\times [0,\infty ) \rightarrow {\mathbb {R}}$ is a positive solution to the heat equation

1.1 $$\begin{aligned} u_t-\Delta _g u=0, \end{aligned}$$

on an *n*-dimensional complete Riemannian manifold $(M^n,g)$ with nonnegative Ricci curvature, then

1.2 $$\begin{aligned} \frac{u_t}{u}-\frac{|\nabla u|^2}{u^2} +\frac{n}{2t} = \Delta \log u +\frac{n}{2t} \ge 0 \end{aligned}$$

for all $(x,t)\in M\times (0,\infty )$. Remarkably, the equality in (1.2) is achieved on the heat kernel on the Euclidean space ${\mathbb {R}}^n$. The Li-Yau estimate is also called a differential Harnack inequality since integrating it yields a sharp version of the classical Harnack inequality originated from Moser [53]. The Li-Yau estimate and its generalizations in various settings provide a versatile tool for studying the analytical, topological, and geometrical properties of manifolds (see for instance the classical books [63] and [37]).

Under the stronger assumption that $(M^n,g)$ has nonnegative sectional curvature and parallel Ricci curvature, Hamilton [30] extended the Li-Yau estimate to the full matrix version

1.3 $$\begin{aligned} \nabla _i \nabla _j \log u +\frac{1}{2t} g_{ij} \ge 0. \end{aligned}$$

Note that the trace of (1.3) is (1.2). Later on, Chow and Hamilton [12] further extended (1.2) and (1.3) to the constrained case under the same curvature assumptions, and discovered new linear Harnack estimates.

When $(M^n,g)$ is a complete Kähler manifold with nonnegative bisectional curvature, Cao and Ni [20] proved the matrix inequality

1.4 $$\begin{aligned} \nabla _\alpha \nabla _{\bar{\beta }} \log u +\frac{1}{t}g_{\alpha \bar{\beta }} \ge 0, \end{aligned}$$

and they called it a matrix Li–Yau–Hamilton estimate. Inspired by Chow’s interpolation consideration [14] of Li-Yau’s and Hamilton’s Harnack inequalities on a surface, Chow and Ni [56, Theorem 2.2] proved that if $(M^n, g(t))$ is a complete solution to Kähler-Ricci flow with bounded nonnegative bisectional curvature and if *u* is a positive solution to the forward conjugate heat equation

1.5 $$\begin{aligned} u_t-\Delta _{g(t)} u=Ru, \end{aligned}$$

then

1.6 $$\begin{aligned} R_{\alpha \bar{\beta }} + \nabla _\alpha \nabla _{{\bar{\beta }}} \log u +\frac{1}{t}g_{\alpha {\bar{\beta }}} \ge 0. \end{aligned}$$

The equality holds if and only if $(M^n,g(t))$ is an expanding Kähler-Ricci soliton. These results were generalized to the constrained case by Ren, Yao, Shen, and Zhang [62].

When the metrics are evolved by the Ricci flow (see [27] or [16])

$$\begin{aligned} \partial _t g =-2\text {Ric}, \end{aligned}$$

Perelman [60] discovered a spectacular differential Harnack estimate for the fundamental solution to the backward conjugate heat equation

1.7 $$\begin{aligned} u_t+\Delta _{g(t)} u =R u, \end{aligned}$$

where *R* denotes the scalar curvature of $(M^n,g(t))$. Astoundingly, his estimate did not require any curvature conditions. For more information on matrix Li–Yau–Hamilton estimates and differential Harnack estimates, as well as their important applications in geometry, we refer the reader to Chow’s survey [15], Ni’s survey [57], and the monographs [10, Chapters 15-16] and [11, Chapters 23-26] by Chow, etc.

In this paper, we first extend Hamilton’s matrix estimate (1.3) for static metrics to the Ricci flow case. Our estimate does not require the parallel Ricci curvature condition and thus should be more applicable.

#### Theorem 1.1

Let $(M^n,g(t))$, $t\in [0,T]$, be a complete solution to the Ricci flow. Let $u:M^n \times [0,T] \rightarrow {\mathbb {R}}$ be a positive solution to the heat equation

1.8 $$\begin{aligned} u_t -\Delta _{g(t)}u=0. \end{aligned}$$

Suppose that $(M^n,g(t))$ has nonnegative sectional curvature and $\text {Ric}\le \kappa g$ for some constant $\kappa >0$. Then

1.9 $$\begin{aligned} \nabla _i \nabla _j \log u + \frac{\kappa }{1-e^{-2\kappa t}} g_{ij} \ge 0, \end{aligned}$$

for all $$(x,t)\in M\times (0,T)$$.

#### Remark 1.1

Note that (1.9) can be restated equivalently as

$$\begin{aligned} \nabla _i \nabla _j u +\frac{\kappa u}{1-e^{-2\kappa t}} g_{ij} +\nabla _i u V_j +\nabla _j u V_i +u V_iV_j \ge 0 \end{aligned}$$

for any vector field *V* by choosing the optimal vector field $V=-\nabla \log u$. Other matrix Li–Yau–Hamilton estimates also admit such equivalent restatements.

#### Remark 1.2

Note that (1.9) is asymptotically sharp as $t\rightarrow 0^+$. To see this, one notices

1.10 $$\begin{aligned} \frac{1}{2t}< \frac{\kappa }{1-e^{-2\kappa t}} < \frac{1}{2t}+\kappa \end{aligned}$$

for all $t>0$ and $\kappa >0$. Applying Theorem 1.1 to $(M^n,g)=({\mathbb {R}}^n,\delta _{ij})$ and letting $\kappa \rightarrow 0^+$ produce $\nabla _i\nabla _j \log u \ge -\frac{1}{2t} \delta _{ij}$, for which the equality is achieved when *u* is the heat kernel on ${\mathbb {R}}^n$.

#### Remark 1.3

For proving the unique continuation result in Corollary 1.8, it is important to obtain a lower bound for $\nabla _i\nabla _j \log u$ that is asymptotic to $\tfrac{1}{2t}g_{ij}$ as $t\rightarrow 0^+$.

Tracing (1.9) yields a gradient estimate.

#### Corollary 1.2

Under the same assumptions as in Theorem 1.1, we have

1.11 $$\begin{aligned} \frac{u_t}{u} -\frac{|\nabla u|^2}{u^2} +\frac{n\kappa }{1-e^{-2\kappa t}} =\Delta \log u +\frac{n\kappa }{1-e^{-2\kappa t}} \ge 0. \end{aligned}$$

On a general compact manifold, Hamilton [30] proved that for any positive solution *u* to (1.1), there exist constants *B* and *C* depending only on the geometry of *M* (in particular the diameter, the volume, and the curvature and covariant derivative of the Ricci curvature) such that $t^{n/2}u\le B$ and

1.12 $$\begin{aligned} \nabla _i \nabla _j \log u +\frac{1}{2t}g_{ij} +C \left( 1+\log \left( \frac{B}{t^{n/2}u}\right) \right) g_{ij} \ge 0. \end{aligned}$$

Here, we also establish such a result for a general compact Ricci flow.

#### Theorem 1.3

Let $(M^n,g(t))$, $t\in [0,T]$, be a compact solution to the Ricci flow. Let $u:M^n \times [0,T] \rightarrow {\mathbb {R}}$ be a positive solution to the heat equation (1.8). Suppose the sectional curvatures of $(M^n,g(t))$ are bounded by *K* for some $K>0$. Then

1.13 $$\begin{aligned} \nabla _i \nabla _j \log u + \left( \frac{1}{2 t} + \frac{1}{t} \beta (t, n, K) \right) g_{ij} \ge 0, \end{aligned}$$

where

$$\begin{aligned} \beta (t, n, K)= 4 \sqrt{n K t} +C_2 (K+1)t +C_1 \sqrt{K} {\text {diam}}. \end{aligned}$$

Here $C_1>0$ is a numerical constant, $C_2>0$ depends only on the dimension and the non-collapsing constant $v_0 = \inf \{|B(x, 1, g(0))|_{g(0)}: x \in M^n \}$, and

$$\begin{aligned} {\text {diam}}:= \sup _{t\in [0,T]} {\text {diam}}(M^n,g(t)). \end{aligned}$$

Compared to (1.12) for static metrics, our estimate (1.13) does not depend on the covariant derivatives of Ricci curvature. We have also made the dependence of the constants on curvature and the diameter more explicit. The proof of Theorem 1.3 is more involved than that of Theorem 1.1, and the key steps are to establish bounds for heat kernel under Ricci flow, $\nabla \log u$, and $\Delta \log u$. In contrast to (1.12) having the term $\log B/u$ whose order is not clear, (1.13) implies a lower bound of $\nabla _i \nabla _j \log u$ that is asymptotic to $-C/t$ as $t\rightarrow 0^+$, where $C=\frac{1}{2}+C_1\sqrt{K}{\text {diam}}$. Therefore, (1.13) can be regarded as a sharp version of Hamilton’s estimate (1.12), just like [72, Theorem 1.1] is a sharp version of the Li-Yau estimate [49, Theorem 1.3]. Finally, we remark that the *C*/*t*-type bound does not hold for noncompact manifolds in general, such as on ${\mathbb {H}}^3$, the three-dimensional hyperbolic space, as explained in [72].

A similar argument yields an improvement of Hamilton’s classical matrix Harnack inequality (1.12) in the static case on compact manifolds.

#### Theorem 1.4

Let $(M^n,g)$ be a closed Riemannian manifold and let $u:M^n \times [0,T] \rightarrow {\mathbb {R}}$ be a positive solution to the heat equation (1.1). Suppose that the sectional curvatures of *M* are bounded by *K* and $|\nabla \text {Ric}|\le L$, for some $K,L>0$. Then

1.14 $$\begin{aligned} \nabla _i \nabla _j \log u + \left( \frac{1}{2t}+(2n-1)K +\frac{\sqrt{3}}{2}L^{\frac{2}{3}} +\frac{1}{2t}\gamma (t,n,K,L)\right) g_{ij} \ge 0 \end{aligned}$$

for all $(x,t)\in M\times (0,T)$, where

$$\begin{aligned} \gamma (t,n,K,L):= & {} \sqrt{nKt(2+(n-1)Kt)} +\sqrt{C_3(K+L^{\frac{2}{3}})t(1+Kt)(1+K+Kt)}\\{} & {} + \left( 2K(2+(n-1)Kt)+\frac{3}{2}L^{\frac{2}{3}}(1+(n-1)Kt) \right) {\text {Diam}}, \end{aligned}$$

$C_3>0$ depends only on the dimension *n*, and ${\text {Diam}}$ denotes the diameter of $(M^n,g)$.

Notice that Hamilton’s original inequality (1.12) has the term $C\log \left( \frac{B}{t^{\frac{n}{2}}u}\right)$, where *B* and *C* depend on the geometry of the manifold and *B* is greater than $t^{\frac{n}{2}}u$, which itself is an additional assumption. The constant *C* is equal to zero only when *M* has nonnegative sectional curvature and parallel Ricci curvature. Otherwise, for this $\log$ term, we do not have any definite control on the order *q* of $-t^{-q}$ coming out of this term, for general positive solutions, making this lower bound less practical. In Theorem 1.4, we manage to replace this term with a *C*/*t* term, with *C* depending only on *K*, *L*, and ${\text {Diam}}$, which is of the correct order for *t*.

Next, we prove a matrix Li–Yau–Hamilton estimate for positive solutions to the backward conjugate heat equation coupled with the Ricci flow.

#### Theorem 1.5

Let $(M^n,g(t))$, $t\in [0,T]$, be a solution to the Ricci flow with nonnegative complex sectional curvature and $\text {Ric}\le \kappa g$ for some $\kappa >0$. Let $u:M^n \times [0,T] \rightarrow {\mathbb {R}}$ be a positive solution to the backward conjugate heat equation (1.7) on $M\times [0,T]$. Suppose $\eta :(0,T) \rightarrow (0,\infty )$ is a $C^1$ function satisfying the ordinary differential inequality

1.15 $$\begin{aligned} \eta ' \le 2\eta ^2 -2\kappa \eta -\frac{\kappa }{t} \end{aligned}$$

on (0, *T*) and that $\eta (t) \rightarrow \infty$ as $t\rightarrow T$. Then

1.16 $$\begin{aligned} R_{ij} -\nabla _i\nabla _j \log u -\eta g_{ij} \le 0 \end{aligned}$$

for all $(x,t) \in M \times (0,T)$, where $R_{ij}$ denotes the Ricci curvature.

#### Remark 1.4

On ancient Ricci flows, we can get rid of the $\frac{\kappa }{t}$ term in (1.15) and prove the cleaner estimate

$$\begin{aligned} R_{ij} -\nabla _i\nabla _j \log u -\frac{\kappa }{1-e^{-2\kappa (T-t)}} g_{ij} \le 0. \end{aligned}$$

See Theorem 6.3 for details.

We give an explicit choice of $\eta (t)$.

#### Corollary 1.6

Under the same assumptions as in Theorem 1.5, we have

1.17 $$\begin{aligned} R_{ij} -\nabla _i\nabla _j \log u -\left( \frac{\kappa }{1-e^{-2\kappa (T-t)}} +\sqrt{\frac{\kappa }{2t}} \right) g_{ij} \le 0. \end{aligned}$$

One motivation for bounding $R_{ij} -\nabla _i\nabla _j \log u$ from above comes from the work of Baldauf and Kim [4]. They defined a parabolic frequency under Ricci flow and proved its monotonicity with a correction factor that depends on the upper bound of $R_{ij} -\nabla _i\nabla _j \log K$, where *K* is the fundamental solution to (1.7). As an application of (1.17), we give an explicit correction factor in Proposition 1.10 in the nonnegative complex sectional curvature case. In addition, we believe such matrix Li–Yau–Hamilton estimates are of their own interest and should be useful in other situations.

We need to assume nonnegative complex sectional curvature in Theorem 1.5 because the proof uses Brendle’s generalization [5] of Hamilton’s Harnack inequality for the Ricci flow [29]. This feature is shared by the proof of (1.6) in [56], which makes use of H.D. Cao’s Harnack estimate for the Kähler-Ricci flow [8]. We also note that it suffices to assume (*M*, *g*(0)) has bounded nonnegative complex sectional curvature, as nonnegative complex sectional curvature is preserved by Ricci flows with bounded curvature (see Brendle and Schoen [6] and Ni and Wolfson [59]).

Finally, we would like to mention that since the pioneer works of Li and Yau [49], Hamilton [30], Perelman [60], and others, various gradient and Hessian estimates for positive solutions to heat-type equations, with either fixed or time-dependent metrics, have been established by many authors, including Guenther [26], Ni [54, 55], Cao and Ni [20], Ni [56], Kotschwar [35], the second author [71], Souplet and the second author [64], Kuang and the second author [36], Cao [9], X. Cao and Hamilton [13], Liu [40], Bailesteanu, X. Cao, and Pulemotov [2], X. Cao and the second author [21], Li and Xu [47], Han and the second author [33], Zhu and the second author [73, 74], Huang [32], and Yu and Zhao [68], just to name a few. Our matrix Li–Yau–Hamilton estimates are new additions to the literature.

### Parabolic frequency

The elliptic frequency

$$\begin{aligned} I_A(r)=\frac{r \int _{B_r(p)} |\nabla u|^2 dx }{\int _{\partial B_r(p)} u^2 dA} \end{aligned}$$

for a harmonic function *u* on ${\mathbb {R}}^n$ was introduced by Almgren [1]. He used its monotonicity to study the local regularity of (multiple-valued) harmonic functions and minimal surfaces. The monotonicity of $I_A(r)$ also played an important role in studying unique continuation properties of elliptic operators by Garafalo and Lin [23, 25] and in estimating the size of nodal sets of solutions to elliptic and parabolic equations by Lin [39]. When ${\mathbb {R}}^n$ is replaced by a Riemannian manifold, Garofalo and Lin [24] proved that there exist constants $R_0$ and $\Lambda$, depending only on the Riemannian metric, such that $e^{\Lambda r}I_A(r)$ is monotone nondecreasing in $(0, R_0)$ (see also Mangoubi [50, Theorem 2.2]). Frequency monotonicity is also crucial in the work of Logunov [43, 44] in estimating the size of nodal sets for harmonic functions and eigenfunctions on manifolds. In addition, frequency functions also play a crucial role in studying the dimension of the space of harmonic functions of polynomial growth on complete noncompact manifolds; see Colding and Minicozzi [17, 18], G. Xu [67], J.Y. Wu and P. Wu [66], Mai and Ou [52], and the references therein. For more applications, we refer the reader to the books [31] and [70].

Poon [61] introduced the parabolic frequency

$$\begin{aligned} I_P(t)=\frac{t \int _{{\mathbb {R}}^n} |\nabla u|^2(x,T-t) G(x,x_0,t)\ dx}{\int _{{\mathbb {R}}^n} u^2(x,T-t) G(x,x_0,t) \ dx}, \end{aligned}$$

where *u* solves the heat equation on ${\mathbb {R}}^n \times [0,T]$ and $G(x,x_0,t)$ is the heat kernel with a pole at $(x_0,0)$. He proved that $I_P(t)$ is monotone nondecreasing and derived some unique continuation results out of it. The monotonicity of $I_P(t)$ remains valid when ${\mathbb {R}}^n$ is replaced by a complete Riemannian manifold with nonnegative sectional curvature and parallel Ricci curvature, as remarked by Poon [61, page 530] and proved independently by Ni [58]. The curvature conditions are needed to use Hamilton’s matrix estimate (1.3).

Without assuming the restrictive parallel Ricci condition, Wang and the first author [45] showed that $t e^{\sqrt{t}}I_P(t)$ is monotone nondecreasing for a short period of time on compact manifolds with nonnegative sectional curvature, which also produces a unique continuation result. They also defined the parabolic frequency

$$\begin{aligned} I_{LW}(t)=\frac{t \int _M |\nabla v(x,t)|^2 R d\mu _{g(t)}}{\int _M v^2(x,t) R d\mu _{g(t)}}, \end{aligned}$$

where *v* solves the backward heat equation $v_t+\Delta _{g(t)} v=0$ coupled with a two-dimensional Ricci flow with positive scalar curvature. Using that *R* satisfies the forward heat equation (1.5) and admits a matrix Li–Yau–Hamilton estimate due to Hamilton (see [16, Proposition 10.20]), they showed that $I_{LW}(t)$ is monotone nondecreasing.

Colding and Minicozzi [19] proved that the parabolic frequency

$$\begin{aligned} I_{CM}(t) = \frac{\int _M |\nabla u(x,t)|^2 e^{-f}d\mu _g}{\int _M u^2(x,t)e^{-f}d\mu _g}, \end{aligned}$$

where *f* is a smooth function on a Riemannian manifold $M^n$ and $u: M^n\times [0,T] \rightarrow {\mathbb {R}}^N$ solves the weighted heat equation $u_t-\Delta _f u=0$, is monotone nonincreasing without any curvature assumptions. The special case $f\equiv 1$ and $M^n$ being a bounded domain in ${\mathbb {R}}^n$ was treated in [22, pages 61-62] to prove the backward uniqueness of the heat equation with specified boundary values. They also defined a parabolic frequency for shrinking gradient Ricci solitons and showed its monotonicity with no curvature restrictions.

For a general Ricci flow, Baldauf and Kim [4] defined the parabolic frequency

$$\begin{aligned} I_{BK}(t):=\frac{(T-t) \int _M |\nabla u(x,t)|^2 K(x,x_0,t) d\mu _{g(t)}}{\int _M u^2(x,t) K(x,x_0,t) d\mu _{g(t)}}, \end{aligned}$$

where *K* is the backward conjugate heat kernel with a pole at $(x_0,T)$ and *u* solves the heat equation (1.8). They were able to show that $e^{\int \frac{1-k(t)}{T-t} dt} \cdot I_{BK}(t)$ is monotone nonincreasing, where *k*(*t*) is a time-dependent function such that

$$\begin{aligned} \text {Ric}-\nabla ^2 \log K \le \frac{k(t)}{2(T-t)}. \end{aligned}$$

More recently, C. Li, Y. Li, and K. Xu [41] studied the monotonicity of $I_{BK}(t)$ and its generalizations under the Ricci flow and the Ricci-harmonic flow. They obtained monotonicity formulas with correction factors depending on the bounds of the Bakry-Émery Ricci curvature or the Ricci curvature. It is also worth mentioning that H.Y. Liu and P. Xu [48] investigated the monotonicity of a parabolic frequency for weighted *p*-Laplacian heat equation with $p\ge 2$ on Riemannian manifolds and obtained generalizations of [19]. Using (1.6), they generalized a frequency monotonicity formula of Ni [58] on Kähler manifolds to the setting of Kähler-Ricci flow.

In this paper, we define a parabolic frequency for solutions to the backward conjugate heat equation (1.7) coupled with the Ricci flow and prove its monotonicity up to certain correction factors. We shall use $G(x,x_0,t)$, the heat kernel with a pole at $(x_0,0)$, as a weight and define the following quantities:

$$\begin{aligned} I(t)= & {} \int _M u^2(x,t) G(x,x_0, t) d\mu _{g(t)}, \\ D(t)= & {} \int _M |\nabla u(x,t)|^2 G(x, x_0,t)d\mu _{g(t)}, \\ S(t)= & {} \int _M u^2(x,t)R(x,t) G(x, x_0,t) d\mu _{g(t)}. \end{aligned}$$

The first two quantities are direct generalizations of the terms in Poon’s parabolic frequency $I_P(t)$ in the static case. The third one is new due to the Ricci flow. A natural generalization of Poon’s frequency $I_P(t)$ is

1.18 $$\begin{aligned} F(t) : =\frac{I'(t)}{I(t)}=\frac{2 D(t) + S(t)}{I(t)}. \end{aligned}$$

In the nonnegative sectional curvature case, we prove that

#### Theorem 1.7

Let $(M^n,g(t))$, $t \in [0,T]$, be a complete Ricci flow and let $u: M^n\times [0,T] \rightarrow {\mathbb {R}}$ be a solution to the backward conjugate heat equation (1.7). Suppose that $(M^n,g(t))$ has nonnegative sectional curvature and $\text {Ric}\le \kappa g$ for some constant $\kappa >0$. Then

1.19 $$\begin{aligned} e^{(n+2)\kappa t}(1-e^{-2\kappa t})F(t), \end{aligned}$$

where *F*(*t*) is defined in (1.18), in monotone nondecreasing on [0, *T*].

We point out that Theorem 1.7 covers, by letting $\kappa \rightarrow 0^+$, Poon’s frequency monotonicity on ${\mathbb {R}}^n$ in [61]. It also implies a unique continuation result (see Lin [38], Poon [61], and Vessella [65] for unique continuation results for parabolic equations).

#### Corollary 1.8

Let $(M^n,g(t))$, $t \in [0,T]$, be a complete Ricci flow with nonnegative sectional curvature and $\text {Ric}\le \kappa g$ for some $\kappa >0$. Suppose that a solution *u*(*x*, *t*) of the backward conjugate heat equation (1.7) on $M^n \times [0,T]$ vanishes of infinite order at $(x_0, t_0) \in M\times (0,T)$, in the sense that

$$\begin{aligned} |u(x,t)| \le O\left( d^2_t(x,x_0) +|t-t_0|\right) ^N \end{aligned}$$

for all positive integer *N* and all (*x*, *t*) near $(x_0,t_0)$. Then $u\equiv 0$ in $M\times [0,T]$.

Under general compact Ricci flows, we prove that *F*(*t*) is monotone up to an implicit correction factor.

#### Theorem 1.9

Let $(M^n,g(t))$, $t \in [0,T]$, be a compact solution to the Ricci flow with sectional curvatures bounded by *K* for some $K>0$. Let $u: M^n \times [0,T] \rightarrow {\mathbb {R}}$ be a solution to the backward conjugate heat equation (1.7). Then, for any $T>0$, there is a power $p=p(T, n, K, v_0, {\text {diam}})>0$ such that

1.20 $$\begin{aligned} t^p (F(t) +Z_0), \end{aligned}$$

where *F*(*t*) is defined in (1.18), is monotone nondecreasing on [0, *T*]. Here $Z_0=Z_0(T, n, K, v_0, {\text {diam}})$ is any sufficiently large number.

Extra curvature terms arise due to the Ricci flow when proving Theorem 1.9. We handle them using some cancellation property and some Li-Yau estimates for the heat kernel under the Ricci flow, together with the matrix Harnack inequality in Theorem 1.3. Besides the above-mentioned results, we also prove the monotonicity of a parabolic frequency without weight at the end of Sect. 5 assuming nonnegative Ricci curvature; see Theorem 5.1.

Finally, we apply Theorem 1.5 to prove that

#### Proposition 1.10

Let $(M^n,g(t))$, $t\in [0,T]$, be a solution to the Ricci flow with nonnegative complex sectional curvature and $\text {Ric}\le \kappa g$ for some $\kappa >0$. Let $u:M^n \times [0,T] \rightarrow {\mathbb {R}}$ be a solution to the heat equation (1.8) and let $w:M^n \times [0,T] \rightarrow (0,\infty )$ be a positive solution to the backward conjugate heat equation (1.7). Then the quantity

1.21 $$\begin{aligned} (e^{2\kappa (T-t)} -1) e^{-\sqrt{8\kappa t}} \cdot \frac{\int _M |\nabla u(x,t)|^2 w(x,t) d\mu _{g(t)}}{\int _M u^2(x,t) w(x,t) d\mu _{g(t)}} \end{aligned}$$

is monotone nonincreasing on [0, *T*].

As mentioned before, Baldauf and Kim [4] proved the monotonicity of $e^{\int \frac{1-k(t)}{T-t} dt}I_{BK}(t)$ under Ricci flow, where *k*(*t*) is a time-dependent function such that

$$\begin{aligned} \text {Ric}-\nabla ^2 \log K \le \frac{k(t)}{2(T-t)}. \end{aligned}$$

However, it is not clear whether such *k*(*t*) exists in the complete noncompact case. In the compact case, the existence of *k*(*t*) is shown by Huang [32] and it depends on |*Rm*|, $|\nabla Rm|$ and $|\nabla ^2 R|$, but no explicit *k*(*t*) is known. Theorem 1.5 and Corollary 1.6 provide an explicit *k*(*t*) in the nonnegative complex sectional curvature case. Proposition 1.10 then gives an explicit correction factor in the monotonicity of $I_{BK}(t)$ and it is also applicable to complete noncompact Ricci flows with bounded nonnegative complex sectional curvature. In addition, we also get a unique continuation result in this case (see Corollary 6.6).

**Note:** Throughout the paper, we assume either *M* is compact or *M* is complete with bounded curvature/geometry, and the functions satisfy certain growth conditions so that the integrals are finite and all integration by parts can be justified.

The rest of this article is organized as follows. In Sect. 2, we derive the evolution equation satisfied by the Hessian of $\log u$, where *u* is a positive solution to heat-type equations. Section 3 deals with the nonnegative sectional case and proves Theorem 1.1. Section 4 gives the proof of Theorem 1.3. Section 5 is devoted to studying the parabolic frequency and proving Theorem 1.7 and Theorem 1.9. In Sect. 6, we prove Theorem 1.5 and Proposition 1.10. In Sect. 7, we prove Theorem 1.4.

## Evolution equations

Let $(M^n,g(t))$, $t\in [0,T]$, be a solution to the Ricci flow. Let $u:M^n\times [0,T] \rightarrow {\mathbb {R}}$ be a positive solution to the heat-type equation

2.1 $$\begin{aligned} (\partial _t -\varepsilon \Delta _{g(t)}) u=\delta Ru, \end{aligned}$$

where $\varepsilon$ and $\delta$ are real parameters. We are mainly interested in the heat equation corresponding to $\varepsilon =1$ and $\delta =0$ and the backward conjugate heat equation corresponding to $\varepsilon =-1$ and $\delta =1$, but the calculations in this section are valid for all $\varepsilon , \delta \in {\mathbb {R}}$.

The main result of this section is the evolution equation satisfied by

$$\begin{aligned} H_{ij} := \nabla _i \nabla _j \log u. \end{aligned}$$

### Proposition 2.1

In the setting described above, we have

2.2 $$\begin{aligned}{} & {} (\partial _t-\varepsilon \Delta )H_{ij} \nonumber \\{} & {} \quad = \delta \nabla _i\nabla _j R + 2 \varepsilon \left( H^2_{ij} +R_{ikjl}\nabla _k v \nabla _l v + \nabla _k H_{ij} \nabla _k v \right) \nonumber \\{} & {} \qquad +\varepsilon (2R_{ikjl}H_{kl}-R_{ik}H_{jk}-R_{jk}H_{ik}) \nonumber \\{} & {} \qquad +(1-\varepsilon ) (\nabla _iR_{jk}+\nabla _jR_{ik}-\nabla _kR_{ij})\nabla _k v, \end{aligned}$$

where $$H^2_{ij}:=H_{ik}H_{jk}$$.

We first prove a commutator formula for $\partial _t-\varepsilon \Delta _L$ and $\nabla _i \nabla _j$, where $\Delta _L$ denotes the Lichnerowicz Laplacian acting on symmetric two-tensors via

$$\begin{aligned} \Delta _L h_{ij} = \Delta h_{ij}+2R_{ikjl}h_{kl}-R_{ik}h_{jk}-R_{jk}h_{ik}. \end{aligned}$$

### Lemma 2.1

Under the Ricci flow, it holds that

2.3 $$\begin{aligned}{} & {} (\partial _t-\varepsilon \Delta _L)(\nabla _i \nabla _j f) \nonumber \\{} & {} \quad \quad = \nabla _i \nabla _j (\partial _t -\varepsilon \Delta ) f + (1-\varepsilon )(\nabla _iR_{jk}+\nabla _jR_{ik}-\nabla _kR_{ij}) \nabla _k f \end{aligned}$$

for any smooth function *f*(*x*, *t*).

### Proof

The cases $\varepsilon =\pm 1$ are proved in [16], so we only do a slight generalization here. The time derivatives of the Christoffel symbols $\Gamma _{ij}^k$ under the Ricci flow are given by (see [16, page 108])

$$\begin{aligned} \partial _t \Gamma _{ij}^k = -g^{kl}(\nabla _i R_{jl} +\nabla _j R_{il} -\nabla _l R_{ij}). \end{aligned}$$

It follows that

$$\begin{aligned} \partial _t(\nabla _i \nabla _j f) = \nabla _i \nabla _j (\partial _t f) +(\nabla _i R_{jk} +\nabla _j R_{ik} -\nabla _l R_{ij})\nabla _k f. \end{aligned}$$

Second, we have

$$\begin{aligned} \Delta _L \nabla _i \nabla _j f =\nabla _i \nabla _j \Delta f +(\nabla _iR_{il}+\nabla _jR_{il}-\nabla _lR_{ij})\nabla _l f. \end{aligned}$$

This can be seen by commuting covariant derivatives as follows

$$\begin{aligned} \nabla _i \nabla _j \Delta f= & {} \nabla _i \nabla _j \nabla _k \nabla _k f \\= & {} \nabla _i \nabla _k \nabla _k \nabla _j f -\nabla _i(R_{jl}\nabla _l f ) \\= & {} \nabla _k \nabla _i \nabla _k \nabla _j f -R_{il}\nabla _l \nabla _j f +R_{ikjl}\nabla _k\nabla _l f \\{} & {} -\nabla _iR_{jl}\nabla _l f -R_{jl}\nabla _i\nabla _l f \\= & {} \nabla _k \nabla _k \nabla _i \nabla _j f +\nabla _k(R_{ikjl}\nabla _l f) -R_{il}\nabla _l \nabla _j f +R_{ikjl}\nabla _k\nabla _l f \\{} & {} -\nabla _iR_{jl}\nabla _l f -R_{jl}\nabla _i\nabla _l f \\= & {} \Delta \nabla _i\nabla _j f +2R_{ikjl}\nabla _k\nabla _l f +\nabla _l R_{ij} \nabla _l f -\nabla _j R_{il} \nabla _l f \\{} & {} -R_{il}\nabla _l \nabla _j f -\nabla _iR_{jl}\nabla _l f -R_{jl}\nabla _i\nabla _l f \\= & {} \Delta _L \nabla _i \nabla _j f -(\nabla _iR_{jl}+\nabla _jR_{il}-\nabla _lR_{ij})\nabla _l f, \end{aligned}$$

where we have used the contracted Bianchi identity

$$\begin{aligned} \nabla _k R_{ikjl} = \nabla _l R_{ij} -\nabla _j R_{il}. \end{aligned}$$

Combining the above two calculations, we obtain (2.3). $\square$

We now prove Proposition 2.1.

### Proof of Proposition 2.1

For convenience, we write $v=\log u$. One derives from (2.1) that *v* satisfies the equation

$$\begin{aligned} (\partial _t -\varepsilon \Delta )v =\varepsilon |\nabla v|^2+\delta R. \end{aligned}$$

We compute that

$$\begin{aligned} \nabla _i\nabla _j |\nabla v|^2= & {} 2\nabla _i (\nabla _j\nabla _k v) \nabla _k v +2 \nabla _i \nabla _k v \nabla _j \nabla _k v \\= & {} 2\nabla _k (\nabla _i\nabla _j v) \nabla _k v +2H^2_{ij} +2R_{ikjl}\nabla _k v \nabla _l v \\= & {} 2H^2_{ij} +2R_{ikjl}\nabla _k v \nabla _l v +2 \nabla _k H_{ij} \nabla _k v, \end{aligned}$$

where $H^2_{ij}:=H_{ik}H_{jk}$. Applying the identity (2.3) to $f=v$ yields

$$\begin{aligned}{} & {} (\partial _t-\varepsilon \Delta _L) H_{ij} \\{} & {} \quad = \nabla _i\nabla _j (\varepsilon |\nabla v|^2+\delta R) \\{} & {} \qquad + (1-\varepsilon ) (\nabla _iR_{jk}+\nabla _jR_{ik}-\nabla _kR_{ij})\nabla _k v \\{} & {} \quad = \delta \nabla _i\nabla _j R + 2\varepsilon \left( H^2_{ij} +R_{ikjl}\nabla _k v \nabla _l v +\nabla _k H_{ij} \nabla _k v \right) \\{} & {} \qquad +(1-\varepsilon ) (\nabla _iR_{jk}+\nabla _jR_{ik}-\nabla _kR_{ij})\nabla _k v. \end{aligned}$$

Finally, (2.2) follows from the above identity and

$$\begin{aligned} \Delta _L H_{ij} = \Delta _L H_{ij} +2R_{ikjl}H_{kl}-R_{ik}H_{jk}-R_{jk}H_{ik}. \end{aligned}$$

The proof is complete. $\square$

## Matrix Harnack for the heat equation

In this section, we prove Theorem 1.1. The proof of Theorem 1.1 divides into two cases: the compact case and the complete noncompact case.

### The compact case

#### Proof of Theorem 1.1

In the compact case, we use Hamilton’s tensor maximum principle to prove Theorem 1.1.

Setting $\varepsilon =1$ and $\delta =0$ in (2.2), we get that $H_{ij}:=\nabla _i\nabla _j \log u$ satisfies

3.1 $$\begin{aligned} (\partial _t-\Delta )H_{ij}= & {} 2H^2_{ij} +(2R_{ikjl}H_{kl}-R_{ik}H_{jk}-R_{jk}H_{ik}) \nonumber \\{} & {} +2R_{ikjl}\nabla _k v \nabla _l v +2\nabla _k H_{ij} \nabla _k v \end{aligned}$$

where $H^2_{ij}:=H_{ik}H_{jk}$. We write

3.2 $$\begin{aligned} c(t):=\frac{\kappa }{1-e^{-2\kappa t}} \end{aligned}$$

and define

$$\begin{aligned} Z_{ij}:=H_{ij}+c(t)g_{ij}. \end{aligned}$$

Direct calculations using (3.1) and the identity

$$\begin{aligned}{} & {} 2H^2_{ij} +2R_{ikjl}H_{kl}-R_{ik}H_{jk}-R_{jk}H_{ik} \\{} & {} \quad = 2Z^2_{ij} -4cZ_{ij} +2c^2 g_{ij} +2R_{ikjl}Z_{kl}-R_{ik}Z_{jk}-R_{jk}Z_{ik} \end{aligned}$$

show that

3.3 $$\begin{aligned} (\partial _t-\Delta )Z_{ij}= & {} 2Z^2_{ij} -4cZ_{ij} +2R_{ikjl}Z_{kl}-R_{ik}Z_{jk}-R_{jk}Z_{ik}\nonumber \\{} & {} +2R_{ikjl}\nabla _k v \nabla _l v +2\nabla _k Z_{ij} \nabla _k v \nonumber \\{} & {} +(c'+2c^2-2\kappa c)g_{ij}+2 c (\kappa g_{ij} -R_{ij}). \end{aligned}$$

Noting that $2R_{ikjl}\nabla _k v \nabla _l v \ge 0$, $R_{ij} \le \kappa g_{ij}$, and *c*(*t*) solves the ODE

$$\begin{aligned} c'=-2c^2 +2\kappa c, \end{aligned}$$

we derive from (3.3) that

3.4 $$\begin{aligned} (\partial _t-\Delta )Z_{ij}\ge & {} 2Z^2_{ij} -4cZ_{ij} +2R_{ikjl}Z_{kl} -R_{ik}Z_{jk}-R_{jk}Z_{ik} \nonumber \\{} & {} + 2\nabla _k Z_{ij} \nabla _k v. \end{aligned}$$

Since *M* is compact and $c(t) \rightarrow \infty$ as $t\rightarrow 0^+$, we have $Z_{ij}\ge 0$ as $t\rightarrow 0^+$. Then the tensor maximum principle of Hamilton [28] implies that $Z_{ij}\ge 0$ for all $t\in [0,T]$, as it is clear that

$$\begin{aligned} 2Z^2_{ij} -4cZ_{ij} +2R_{ikjl}Z_{kl}-R_{ik}Z_{jk}-R_{jk}Z_{ik} \end{aligned}$$

is nonnegative at a null-eigenvector of $Z_{ij}$. The proof is complete. $\square$

### The complete noncompact case

Now we deal with the case that $(M^n, g(t))$, $t\in [0,T]$, is a complete noncompact Ricci flow with nonnegative sectional curvature and $\text {Ric}\le \kappa g$. We note that the uniqueness of solutions to the heat equation (1.8) fails to be true on a complete noncompact manifold. In order to apply Hamilton’s tensor maximum principle (see for instance [10, Theorem 12.33] for a version on complete noncompact Ricci flows) to $Z_{ij}$, one needs to impose some growth condition on the function *u* and its first and second derivatives. Using an idea in [20] and [56], we can, however, get away without assuming any growth conditions on *u*. The key is that we are working with a positive solution of the heat equation and we can make use of the Li-Yau estimate for *u* under the Ricci flow (see [11, Theorem 25.9 and Corollary 25.13]) to obtain required growth estimates at any positive time. Then, we can get integral bounds on the first and second derivatives of *u* via integration by parts. Finally, we use the idea of working with the smallest eigenvalue of the symmetric two-tensor $tuZ_{ij}$ to use a maximum principle for scalar heat equation (see [10, Theorem 12.22]), avoiding the tensor maximum principle which requires a more restrictive growth condition.

We first prove a growth estimate for *u* on a slightly smaller time interval using the Li-Yau estimate and its resulting Harnack inequality.

#### Lemma 3.1

Let $(M^n,g(t))$, $t\in [0,T]$, be a complete solution of the Ricci flow with $-\kappa g \le \text {Ric}\le \kappa g$. Let *u* be a positive solution to the heat equation 1.8 on $M\times [0,T]$. Fix $p\in M$. For any $\delta \in (0, T/3)$, there exist a positive constant $A_1$, depending on $n,\kappa , T, \delta$, and *u*(*p*, *T*) such that

3.5 $$\begin{aligned} u(x,t) \le \exp {\left( A_1(d^2_0(x,p) +1)\right) } \end{aligned}$$

for all $x\in M$ and $t \in [\delta , T-\delta ]$, where $d_0(\cdot ,\cdot )$ is the distance function with respect to *g*(0).

#### Proof

Since $-\kappa g \le \text {Ric}\le \kappa g$, we have

$$\begin{aligned} e^{-2\kappa T} g(0) \le g(t) \le e^{2\kappa T} g(0) \end{aligned}$$

for all $t\in [0,T]$. In the Li-Yau estimate stated in [11, Theorem 25.9] and the Harnack inequality stated in [11, Corollary 25.13], we can take ${\tilde{g}}=g(0)$, ${\tilde{C}}_0=e^{2\kappa T}$, $Q=0$, and $\varepsilon =\tfrac{1}{3}$ and let $R\rightarrow \infty$. Then, we conclude that there exist positive constants $B_1=B_1(n,\kappa )$ and $B_2=B_2(\kappa , T)$, such that

3.6 $$\begin{aligned} \frac{u(x_2,t_2)}{u(x_1,t_1)} \ge e^{-B_1(t_2-t_1)}\left( \frac{t_2}{t_1}\right) ^{-n}\exp {\left( -B_2\frac{d^2_{0}(x_1,x_2)}{t_2-t_1}\right) } \end{aligned}$$

for any $x_1,x_2 \in M$ and $0<t_1 <t_2 \le T$. Applying (3.6) with $x=x_1$, $x_2=p$, $t_1=t$, and $t_2=T$, we get

3.7 $$\begin{aligned} u(x,t) \le u(p,T)e^{B_1(T-t)}\left( \frac{T}{t}\right) ^{n}\exp {\left( B_2\frac{d^2_{0}(x,p)}{T-t}\right) } \end{aligned}$$

for all $(x,t)\in M\times (0,T)$. This implies that, for $(x,t) \in M \times [\delta , T-\delta ]$, there exists a constant $A_1$ depending on $n,\kappa , T, \delta$, and *u*(*p*, *T*) such that (3.5) holds. $\square$

Next, we obtain integral bounds for the first and second derivatives of *u*.

#### Lemma 3.2

Let $(M^n,g(t))$ and *u* be the same as in Lemma 3.1. Assume further that $(M^n,g(t))$ has nonnegative scalar curvature. Then there exists $A_2 \ge A_1$ such that

3.8 $$\begin{aligned} \int _\delta ^{T-\delta } \int _M |\nabla u(x,t)|^2 \exp {\left( -A_2 (d^2_0(x,p)+1) \right) } dx dt < \infty \end{aligned}$$

and

3.9 $$\begin{aligned} \int _\delta ^{T-\delta } \int _M |\nabla ^2 u(x,t)|^2 \exp {\left( -A_2 (d^2_0(x,p)+1) \right) } dx dt < \infty . \end{aligned}$$

#### Proof

We derive from $u_t=\Delta u$ that

$$\begin{aligned} (\partial _t-\Delta )u^2 =-2|\nabla u|^2. \end{aligned}$$

Multiplying both sides by a cut-off function $\varphi ^2$ (independent of time) and integrating by parts yield

$$\begin{aligned}{} & {} 2 \int _\delta ^{T-\delta } \int _M \varphi ^2 |\nabla u|^2 dx dt \\{} & {} \quad = -\int _\delta ^{T-\delta } \int _M \varphi ^2 (\partial _t -\Delta ) u^2 dx dt\\{} & {} \quad \le \int _M \varphi ^2 u^2(x,\delta ) dx +4 \int _\delta ^{T-\delta } \int _M \varphi u |\nabla \varphi | |\nabla u| dx dt \\{} & {} \quad \le \int _M \varphi ^2 u^2(x,\delta ) dx +4 \int _\delta ^{T-\delta } \int _M |\nabla \varphi |^2 u^2 dx dt \\{} & {} \qquad + \int _\delta ^{T-\delta } \int _M \varphi ^2 |\nabla u| ^2 dx dt. \end{aligned}$$

Now (3.8) follows from (3.5). Applying the same argument to

$$\begin{aligned} (\partial _t-\Delta )|\nabla u|^2 =2 |\nabla ^2 u|^2 \end{aligned}$$

produces (3.9). $$\square $$

#### Proof of Theorem 1.1: the complete noncompact case

Recall that

$$\begin{aligned} Z_{ij}:=H_{ij}+c(t)g_{ij}, \end{aligned}$$

where $c(t)=\frac{\kappa }{1-e^{-2\kappa t}}$, satisfies (3.4) under our curvature assumptions. Define

$$\begin{aligned} {\widetilde{Z}}_{ij} :=tuZ_{ij}. \end{aligned}$$

Using (3.4), we derive that

3.10 $$\begin{aligned} (\partial _t -\Delta ) {\widetilde{Z}}_{ij}\ge & {} \tfrac{1}{t}{\widetilde{Z}}_{ij} +\tfrac{2}{tu}{\widetilde{Z}}^2_{ij}-4c(t){\widetilde{Z}}_{ij} \nonumber \\{} & {} +2R_{ikjl}{\widetilde{Z}}_{kl}-R_{ik}{\widetilde{Z}}_{jk}-R_{jk}{\widetilde{Z}}_{ik}. \end{aligned}$$

Next, let’s consider the function $\alpha (x,t)$ on $M\times [0,T]$ defined by

$$\begin{aligned} \alpha (x,t) =\inf \{s \ge 0: {\widetilde{Z}}_{ij}(x,t)+sg_{ij}(x,t) \ge 0 \}. \end{aligned}$$

In other words,

3.11 $$\begin{aligned} \alpha (x,t)=\max \{ 0, -\lambda _1(x,t)\} \end{aligned}$$

on $M\times (0,T)$, where $\lambda _1(x,t)$ is the smallest eigenvalue of ${\widetilde{Z}}_{ij}$ at (*x*, *t*). The key is to show that

3.12 $$\begin{aligned} (\partial _t-\Delta )\alpha \le 0 \end{aligned}$$

holds in the following barrier sense: for any $(x,t)\in M \times (0, T)$, we can find a neighborhood $U \subset M \times (0, T)$ of (*x*, *t*) and a smooth (lower barrier) function $\phi : U \rightarrow {\mathbb {R}}$ such that $\phi \le \alpha$ on *U*, with equality at (*x*, *t*), and

3.13 $$\begin{aligned} (\partial _t-\Delta )\phi \le 0 \end{aligned}$$

at (*x*, *t*). Note that inequality (3.12) holds also in the viscosity sense and in the sense of distributions by standard arguments (see [51] for an elliptic version).

To prove (3.12), we consider $Y_{ij}(x,t):={\widetilde{Z}}_{ij}(x,t)+\alpha (x,t)g_{ij}(x,t)$. Fix $(x,t)\in M\times (0,T)$. By the definition of $\alpha (x,t)$, we have $Y_{ij}\ge 0$ on $M\times [0,T]$ and there exists a unit vector $e_1 \in T_xM$ such that $Y(e_1,e_1)=0$. We extend $e_1$ to an orthonormal basis $\{e_i\}_{i=1}^n$ of $T_xM$ consisting of eigenvector of ${\widetilde{Z}}_{ij}$ such that ${\widetilde{Z}}(e_i)=\lambda _i e_i$ with $\lambda _1 \le \cdots \le \lambda _n$. Next, we extend $\{e_i\}_{i=1}^n$ smoothly in a neighborhood *U* of (*x*, *t*) by parallel translation along radial geodesics using $\nabla ^{g(t)}$ and regard the resulting vector fields, still denoted by $\{e_i\}_{i=1}^n$, as stationary in time in the sense that $\partial _t e_i=0$ for each $1\le i \le n$.

If $\lambda _1(x,t)>0$, then $\alpha \equiv 0$ near (*x*, *t*) and the barrier function $\phi \equiv 0$ will fulfill (3.13). If $\lambda _1(x,t)\le 0$, then the function $\phi (x,t)=-\frac{{\widetilde{Z}}(e_1,e_1)}{g(t)(e_1,e_1)}$ is defined in *U* and gives a lower barrier for $\alpha (x,t)$ in that neighborhood. Therefore, we get using (3.10) that at (*x*, *t*),

$$\begin{aligned} (\partial _t-\Delta )\phi= & {} -(\partial _t-\Delta ) \frac{{\widetilde{Z}}(e_1,e_1)}{g(t)(e_1,e_1)}\\= & {} -\tfrac{1}{t}\lambda _1 -\tfrac{2}{tu}\lambda _1^2 + 4c(t)\lambda _1 - 2R_{1k1k}\lambda _k \\\le & {} \tfrac{1}{t}\alpha -\tfrac{2}{tu}\alpha ^2 -4c(t)\alpha + 2\kappa \alpha \\\le & {} \alpha \left( \tfrac{1}{t}-4c(t)+2\kappa \right) \\\le & {} 0, \end{aligned}$$

where we have used (3.11), the estimate

$$\begin{aligned} R_{1k1k}\lambda _k = R_{1k1k}(\lambda _k-\lambda _1) +R_{11}\lambda _1 \ge -\alpha R_{11} \ge -\alpha \kappa , \end{aligned}$$

and the elementary inequality

$$\begin{aligned} \tfrac{1}{t}-4c(t)+2\kappa < 0 \text { for } t>0. \end{aligned}$$

Hence, we have proved $(\partial _t-\Delta )\alpha \le 0$ in the barrier sense.

Without loss of generality, we may assume $u\ge \varepsilon >0$. This is because once the estimate has been established for $u_\varepsilon :=u+\varepsilon$, one can then let $\varepsilon \rightarrow 0$ and get the estimate for any positive *u*. By shifting the time from *t* to $t+\delta$, we have the growth bounds (3.5), (3.8), and (3.9), which implies that there exists $b>0$ such that

3.14 $$\begin{aligned} \int _0^T \int _M \exp {\left( -b d^2_0(x,p) \right) } \alpha ^2(x,t) dx dt < \infty , \end{aligned}$$

Since $\alpha (x,0)=0$ for all $x\in M$, we can use the maximum principle (see [10, Theorem 12.22]) to conclude that $\alpha (x,t)\le 0$ on $M\times [0,T]$. $\square$

## Matrix Harnack for the heat equation: the general case

In this section, we prove Theorem 1.3. Without the nonnegativity of sectional curvatures, we have to estimate the terms involving curvature and derivatives of *u* and the proof becomes much more involved. Here we employ an idea that has has been used in [46, 69], and [72], namely we first prove the estimate for the heat kernel and then derive the estimate for any positive solution to the heat equation.

### Proof of Theorem 1.3

The proof is divided into three steps.

*Step 1.* We derive a partial differential inequality satisfied by the smallest eigenvalue of $Q_{ij}:=t H_{ij}$, where $H_{ij} = \nabla _i \nabla _j \log u$ as before.

A straightforward computation using (3.1) shows that $Q_{ij}$ satisfies

4.1 $$\begin{aligned} (\partial _t-\Delta )Q_{ij}= & {} \frac{1}{t}Q_{ij} +\frac{2}{t}Q^2_{ij} +2R_{ikjl}Q_{kl}-R_{ik}Q_{jk}-R_{jk}Q_{ik} \nonumber \\{} & {} +2tR_{ikjl}\nabla _k v \nabla _l v +2\nabla _k Q_{ij} \nabla _k v. \end{aligned}$$

Here and through out this section $v =\log u$.

Let $\lambda _1$ be the minimum negative eigenvalue of $Q_{ij}$ in $M^n \times [0, t_0]$ which is reached at the point $(x_0, t_0)$. Note that we are done with the proof if no such $\lambda _1$ exists. Our task is to find a lower bound for $\lambda _1$. Let $\eta$ be a unit eigenvector with respect to the metric $g(t_0)$ at $x_0$. Using parallel transport, we extend $\eta$ along geodesic rays starting from $x_0$ so that it becomes a parallel unit vector field in a neighborhood of $x_0$ with respect to $g(t_0)$. This vector field, still denoted by $\eta =\eta (x)$, is regarded as stationary in the time interval $[0, t_0]$. Now consider the vector field

$$\begin{aligned} \xi =\xi (x, t) = \frac{\eta (x)}{\Vert \eta (x) \Vert _{g(x, t)}} \end{aligned}$$

which is a unit one with respect to *g*(*t*). In local coordinates, we write $\xi =(\xi _1, \ldots , \xi _n)$ and we also introduce the scalar function

$$\begin{aligned} \Lambda =\Lambda (x, t) = \xi _i Q_{ij} \xi _j =\xi ^T (Q_{ij}) \xi . \end{aligned}$$

Notice that $\Lambda$ is a smooth function defined in a neighborhood of $x_0$ on the time interval $[0, t_0]$ and reaches its minimum value $\lambda _1$ at the point $(x_0, t_0)$. Using (4.1), we find that

$$\begin{aligned}{} & {} \xi _i [(\partial _t-\Delta )Q_{ij} ] \xi _j\\{} & {} \quad = \frac{1}{t}\xi _i Q_{ij} \xi _j +\frac{2}{t}\xi _i Q^2_{ij} \xi _j +2R_{ikjl}Q_{kl}\xi _i \xi _j-R_{ik}Q_{jk} \xi _i \xi _j-R_{jk}Q_{ik} \xi _i \xi _j \\{} & {} \qquad +2tR_{ikjl}\nabla _k v \nabla _l v \xi _i \xi _j + 2\nabla _k Q_{ij} \nabla _k v \xi _i \xi _j. \end{aligned}$$

Recall that that at $t=t_0$, $\xi$ is a parallel vector field and

$$\begin{aligned} \partial _t (\xi _i Q_{ij} \xi _j)= \partial _t \left( \frac{\eta _i Q_{ij} \eta _j}{g_{ij} \eta _i \eta _j}\right) = \xi _i \partial _t Q_{ij} \xi _j + 2 \xi _i Q_{ij} \xi _j R_{kl} \xi _k \xi _l. \end{aligned}$$

Combining the above two identities, we deduce, at $(x,t)=(x_0, t_0)$, that

4.2 $$\begin{aligned} (\partial _t-\Delta ) \Lambda= & {} \frac{1}{t} \Lambda +\frac{2}{t} \Lambda ^2 +2R_{ikjl}Q_{kl}\xi _i \xi _j \nonumber \\{} & {} +2tR_{ikjl}\nabla _k v \nabla _l v \xi _i \xi _j + 2\nabla _k \Lambda \nabla _k v. \end{aligned}$$

Notice the terms involving the Ricci curvature are canceled. We remark that this equation may not be satisfied for $t<t_0$ but the proof uses this equation only at $(x,t)=(x_0, t_0)$. As mentioned, $\Lambda$ reaches its minimum value at $(x_0, t_0)$. Therefore, (4.2) implies, at $(x_0, t_0)$

4.3 $$\begin{aligned} 2 \lambda ^2_1 \le - \lambda _1 - 2 t R_{ikjl}Q_{kl}\xi _i \xi _j - 2t^2 R_{ikjl}\nabla _k v \nabla _l v \xi _i \xi _j. \end{aligned}$$

Next, we aim to bound the curvature terms on the right-hand side of (4.3). First, we write

$$\begin{aligned} 2R_{ikjl}Q_{kl}= & {} 2(R_{ikjl}+K(g_{ij}g_{kl}-g_{il}g_{jk}))Q_{kl} -2K(g_{ij}g_{kl}-g_{il}g_{jk})Q_{kl}\\:= & {} 2 w_{ikjl} Q_{kl} -2K(g_{ij}g_{kl}-g_{il}g_{jk})Q_{kl}. \end{aligned}$$

Besides the lowest negative eigenvalue $\lambda _1$, let $\lambda _2$, ..., $\lambda _n$ be other eigenvalues of $(Q_{ij})$ at $(x_0, t_0)$ arranged in increasing order. After diagonalizing $(Q_{ij})$ at $(x_0, t_0)$ with an orthonormal basis $\{\xi , \ldots \}$, we deduce

$$\begin{aligned} R_{ikjl}Q_{kl} \xi _i \xi _j= & {} \sum _k w_{1k1k} \lambda _k -K(g_{ij}g_{kl}-g_{il}g_{jk})Q_{kl} \xi _i \xi _j \\= & {} \sum _{\lambda _k \ge 0} w_{1k1k} \lambda _k + \sum _{\lambda _k< 0} \, w_{1k1k} \lambda _k - K t \Delta v + K \lambda _1\\\ge & {} \sum _{\lambda _k < 0} \, w_{1k1k} \lambda _1 -K t \Delta v +K \lambda _1. \end{aligned}$$

Here we just used the assumption on sectional curvature

$$\begin{aligned} R_{ikjl}\ge -K(g_{ij}g_{kl}-g_{il}g_{jk}) \end{aligned}$$

or $w_{ikjl}\ge 0$ and the identity

$$\begin{aligned} {\text {tr}}(Q_{ij})= t \Delta v. \end{aligned}$$

Using the upper bound of the sectional curvature

$$\begin{aligned} R_{ikjl}\le K(g_{ij}g_{kl}-g_{il}g_{jk}), \end{aligned}$$

we see that

$$\begin{aligned} \sum _k w_{1k1k} \le 2K \sum _k (g_{11}g_{kk}-g_{1k}g_{1k})= 2K (n-1) \end{aligned}$$

and we arrive at, via $\lambda _1<0$, that

4.4 $$\begin{aligned} R_{ikjl}Q_{kl} \xi _i \xi _j \ge (2n-1) K \lambda _1 -K t \Delta v. \end{aligned}$$

Using the lower bound on the sectional curvatures again, noticing $\xi =(1, 0, \ldots , 0)$ and $g_{ij} =\delta _{ij}$ at $(x_0, t_0)$ by our choice of the orthonormal coordinates, we have that

4.5 $$\begin{aligned} R_{ikjl}\nabla _k v \nabla _l v \xi _i \xi _j= & {} R_{1k1l} \nabla _k v \nabla _l v \nonumber \\\ge & {} -K (g_{kl}-g_{1l}g_{1k}) \nabla _k v \nabla _l v \nonumber \\= & {} -K|\nabla v|^2 + K |\nabla _1 v|^2 \nonumber \\\ge & {} -K|\nabla v|^2. \end{aligned}$$

Substituting (4.4), (4.5) into (4.3), we deduce, for $v = \log u$,

4.6 $$\begin{aligned} 2 \lambda ^2_1 \le - \lambda _1 - 2 t (2n-1) K \lambda _1 + 2 K t^2 \Delta v + 2 t^2 K|\nabla v|^2. \end{aligned}$$

*Step 2.* We need to bound the right hand side of (4.6). This might be difficult for all positive solutions *u* but doable for the heat kernel.

In this step, we assume $u(x,t)=G(x,t,y):=G(x, t; y, 0)$ is the heat kernel with a pole at $y\in M, t=0$ and $v=\log u$. It is known that the following curvature-free bound holds:

4.7 $$\begin{aligned} (t-(t_0/2))^2 |\nabla \log u|^2 \le (t-(t_0/2)) \log \frac{\sup \{u(x,t) : (x,t) \in M \times [t_0/2,t_0] \}}{u} \end{aligned}$$

for all $(x,t) \in M \times [t_0/2,t_0]$ (see [71] or [13]). Under the condition of bounded sectional curvature, the upper and lower bound for the heat kernel can be obtained in a classical way in any finite time interval and hence are more or less known. By now, we know that only the pointwise bound on the scalar curvature and initial volume non-collapsing condition are needed for the heat kernel bounds to hold (see [7, Theorem 1.4]). Using that theorem repeatedly over fixed time intervals and taking advantage of the reproducing formula of the heat kernel, we know that the following bounds hold: there exists a numerical positive constant $C_1$ and another positive constant $C_2$ depending only on the volume non-collapsing constant of $g_0$ and the dimension such that

4.8 $$\begin{aligned} \frac{1}{C_2 t^{n/2}} e^{ -C_2 K t- C_1 d^2(x, y, t)/t} \le G(x, t, y) \le \frac{C_2}{t^{n/2}} e^{ C_2 K t-d^2(x, y, t)/(C_1 t)}. \end{aligned}$$

Alternatively, since the sectional curvature is bounded, one can just follow the classical method by Li-Yau to obtain such bounds. The above bounds are far from optimal for large times. Since the manifold is compact, the large-time behavior of the heat kernel is relatively simple since positive solutions tend to be constant. But we will not pursue an optimal large-time bound this time.

Substituting (4.8) to (4.7), we obtain, for all $t_0>0$

4.9 $$\begin{aligned} t^2_0 |\nabla \log u|^2(x, t_0) \le C_3 (K+1) t^2_0+ 2 C_1 \sup _{t \in [t_0/2, t_0]} d^2(x, y, t). \end{aligned}$$

Here $C_3$ depends only on the volume noncollapsing constant of $g_0$ and the dimension.

Next, we need to find an upper bound for the term $t^2 \Delta \log u= t^2(\frac{\Delta u}{u} - \frac{|\nabla u|^2}{u^2})$. In the stationary case, this is done in Hamilton [30, Lemma 4.1]. Following that proof, the Ricci flow produces one extra term involving the Ricci curvature. Since the sectional curvature is bounded, we can treat this term without much difficulty.

Let *L* be the operator

4.10 $$\begin{aligned} L = \Delta + 2 \nabla \log u \nabla - \partial _t. \end{aligned}$$

The following identities are well known and also follow from the calculations in Sect. 2.

4.11 $$\begin{aligned} L (\Delta u/u) = 2 R_{ij} \nabla _i \nabla _j u / u, \quad L (|\nabla \log u|^2) = 2|\nabla _i \nabla _j \log u |^2. \end{aligned}$$

Therefore,

$$\begin{aligned}{} & {} L \left( \frac{\Delta u}{u} + |\nabla \log u|^2 \right) \\{} & {} \quad = 2|\nabla _i \nabla _j \log u |^2 - 2 R_{ij} \left( \frac{\nabla _i\nabla _j u}{u} - \frac{\nabla _i u \nabla _j u}{u^2} \right) - 2 R_{ij} \frac{\nabla _i u \nabla _j u}{u^2}\\{} & {} \quad = 2|\nabla _i \nabla _j \log u |^2 - 2 R_{ij} \nabla _i \nabla _j \log u - 2 R_{ij} \frac{\nabla _i u \nabla _j u}{u^2}\\{} & {} \quad =|\nabla _i \nabla _j \log u |^2 + | \nabla _i \nabla _j \log u - R_{ij} |^2 - R^2_{ij}- 2 R_{ij} \frac{\nabla _i u \nabla _j u}{u^2}\\{} & {} \quad \ge \frac{1}{n} |\Delta \log u|^2 + \frac{1}{n} |\Delta \log u -R|^2 - C_n K^2 - C_n K |\nabla \log u|^2. \end{aligned}$$

Here, $C_n$ is a dimensional constant and the assumption $|R_{ijkl}| \le K (g_{ik}g_{jl}-g_{il}g_{jk})$ has been used. Writing

$$\begin{aligned} Y= \frac{\Delta u}{u} + |\nabla \log u|^2, \end{aligned}$$

then the above inequality implies

$$\begin{aligned} L Y\ge & {} \frac{1}{n} \left| Y- 2|\nabla \log u|^2 \right| ^2 + \frac{1}{n} \left| Y- 2|\nabla \log u|^2 -R \right| ^2 \\{} & {} - C_n K^2 - C_n K |\nabla \log u|^2. \end{aligned}$$

Therefore,

4.12 $$\begin{aligned} L (t^2 Y)\ge & {} \frac{1}{n t^2} \left| t^2 Y- 2 t^2|\nabla \log u|^2 \right| ^2 \nonumber \\{} & {} + \frac{1}{n t^2} \left| t^2 Y- 2 t^2|\nabla \log u|^2 - t^2 R \right| ^2 \nonumber \\{} & {} - C_n t^2 K^2 - C_n K t^2 |\nabla \log u|^2 - 2 \frac{t^2 Y}{t}. \end{aligned}$$

Let $T>0$ be any fixed time and $(x_0, t_0)$ be a maximal point of $t^2 Y$ in $M^n \times (0, T]$ where $t^2 Y$ reaches a positive maximum value. Note that $t_0$ may be less than *T* but the argument by maximum principle together with (4.9) is good enough to bound $t^2 Y$ up to *T* and hence for all time. By (4.12), we know, at $(x_0, t_0)$, the following inequality holds:

$$\begin{aligned} \frac{1}{n t^2} \left| t^2 Y- 2 t^2|\nabla \log u|^2 \right| ^2 \le C_n t^2 K^2 + C_n K t^2 |\nabla \log u|^2 + 2 \frac{t^2 Y}{t}. \end{aligned}$$

Hence for all $t \in (0, T]$, we have

$$\begin{aligned} \frac{1}{2 n t^2} |t^2 Y|^2 \le \left( \frac{1}{n t^2} |2 t^2|\nabla \log u|^2 |^2 + C_n t^2 K^2 + C_n K t^2 |\nabla \log u|^2 + 2 \frac{t^2 Y}{t} \right) _{(x_0, t_0)}. \end{aligned}$$

Now we take *u* to be the heat kernel *G*(*x*, *t*, 0). Using (4.9) we conclude that

$$\begin{aligned} t^2 Y \le 4n t + C_3 (K+1) t^2 + 2 C_1 {\text {diam}}^2 \end{aligned}$$

which yields, since $Y= \frac{\Delta u}{u} + |\nabla \log u|^2$, that

4.13 $$\begin{aligned} t^2 \frac{\Delta u}{u} \le 4nt + C_3 (K+1) t^2 + 2 C_1 {\text {diam}}^2. \end{aligned}$$

From (4.6) and the relation $\Delta v = \Delta \log u =\frac{\Delta u}{u} - |\nabla \log u|^2$, we see that

4.14 $$\begin{aligned} 2 \lambda ^2_1 \le - \lambda _1 - 2 t (2n-1) K \lambda _1 + 2 K t^2 \frac{\Delta u}{u}. \end{aligned}$$

Using (4.13), we deduce that

4.15 $$\begin{aligned} 2 \lambda ^2_1\le & {} - \lambda _1 - 2 t (2n-1) K \lambda _1 \nonumber \\{} & {} + 2 K [4nt + C_3 (K+1) t^2 + 2 C_1 {\text {diam}}^2]. \end{aligned}$$

Since $\lambda _1<0$ by assumption, we conclude, after elementary estimates,

4.16 $$\begin{aligned} t (H_{ij})\ge & {} \lambda _1 (g_{ij}) \nonumber \\\ge & {} \left( - \frac{1}{2} -4 \sqrt{nK t} -C_2 (K+1)t -C_1 \sqrt{K} {\text {diam}}\right) (g_{ij})\nonumber \\:= & {} \left( - \tfrac{1}{2} -\beta (t, n, K, {\text {diam}})\right) (g_{ij}), \end{aligned}$$

where $C_1$ is a numerical constant and $C_2$ depends only on the non-collapsing constant $v_0$ of $g_0$ and the dimension. Note that we have renamed $C_3$ to $C_2$ for consistency with the statement of the theorem.

*Step 3.* Finally, we show the matrix Harnack estimate holds for any positive solution *u*(*x*, *t*) to the heat equation.

Note that

$$\begin{aligned} u(x,t) =\int _M G(x,t,y)u_0(y) dy. \end{aligned}$$

Differentiating under the integral yields

$$\begin{aligned} \nabla _j u(x, t)= & {} \int _M \nabla _j G(x,t,y) u_0(y) dy, \\ \nabla _i \nabla _j u(x, t)= & {} \int _M \nabla _i \nabla _j G(x,t,y) u_0(y) dy. \end{aligned}$$

Here and later in the step $dy=dg(0)(y)$ etc. Therefore,

$$\begin{aligned}{} & {} t u^2 \nabla _i\nabla _j \log u(x, t) \\{} & {} \quad =t ( u \nabla _i \nabla _j u -\nabla _i u \nabla _j u)(x, t) \\{} & {} \quad = \int _M t G(x,t,z) u_0(z) dz \int _M \nabla _i \nabla _j G(x,t,y) u_0(y) dy \\{} & {} \qquad -\int _M t \nabla _j G(x,t,z) u_0(z) dz \int _M \nabla _i G(x,t,y) u_0(y) dy. \end{aligned}$$

Hence,

4.17 $$\begin{aligned}{} & {} t u^2 \nabla _i\nabla _j \log u(x, t) \nonumber \\{} & {} \quad = \int _M \int _M t G(x,t,z) \nabla _i \nabla _j G(x,t,y) u_0(z) u_0(y) dzdy \nonumber \\{} & {} \qquad -\int _M \int _M t \nabla _j G(x,t,z) \nabla _i G(x,t,y) u_0(z) u_0(y) dzdy. \end{aligned}$$

Fixing a space time point (*x*, *t*), $t>0$. Let us diagonalize $(Q_{ij})=t (\nabla _i\nabla _j\log u(x, t))$ using its orthonormal eigenvectors $\{e_1, \ldots , e_n \}$ such that $e_1$ corresponds to the smallest eigenvalue $\lambda _1$. By (4.17), we have

4.18 $$\begin{aligned}{} & {} t u^2 \nabla _1\nabla _1 \log u(x, t) \nonumber \\{} & {} \quad = \int _M \int _M t G(x,t,z) \nabla _1 \nabla _1 G(x,t,y) u_0(z) u_0(y) dzdy \nonumber \\{} & {} \qquad -\int _M \int _M t \nabla _1 G(x,t,z) \nabla _1 G(x,t,y) u_0(z) u_0(y) dzdy. \end{aligned}$$

According to (4.16) in Step 2, the following holds

$$\begin{aligned} t \left( \frac{\nabla _1 \nabla _1 G(x,t,y)}{G(x, t, y)} - \frac{|\nabla _1 G(x,t,y)|^2}{G^{2}(x, t, y)}\right) \ge -\frac{1}{2}-\beta (t, n, K) \end{aligned}$$

so that

$$\begin{aligned} t \nabla _1 \nabla _1 G(x,t,y)\ge & {} t \frac{|\nabla _1 G(x,t,y)|^2}{G(x, t, y)} \\{} & {} -\left( \tfrac{1}{2}+\beta (t, n, K)\right) G(x, t, y). \end{aligned}$$

Substituting the last inequality into (4.18) and regrouping the third term on the right-hand side, we deduce

4.19 $$\begin{aligned}{} & {} t u^2 \nabla _1\nabla _1 \log u(x, t) \nonumber \\{} & {} \quad \ge \int _M \int _M G(x,t,z) t \frac{|\nabla _1 G(x,t,y)|^2}{G(x, t, y)} u_0(z) u_0(y) dzdy \nonumber \\{} & {} \qquad - [\frac{1}{2}+\beta (t, n, K)] \int _M \int _M G(x,t,z) G(x,t,y) u_0(z) u_0(y) dzdy \nonumber \\{} & {} \qquad -\int _M \int _M \frac{ \sqrt{t} \nabla _1 G(x,t,z) \sqrt{G(x,t,y)}}{ \sqrt{G(x,t,z)}} \sqrt{u_0(z) u_0(y)} \nonumber \\{} & {} \qquad \times \frac{ \sqrt{t} \nabla _1 G(x,t,y) \sqrt{G(x,t,z)}}{ \sqrt{G(x,t,y)}} \sqrt{u_0(z) u_0(y)} dzdy. \end{aligned}$$

Observe that the first term on the right-hand side dominates the third term due to the Cauchy-Schwarz inequality and the integral in the second term is $u^2(x,t)$. Hence we have proven

$$\begin{aligned} t \nabla _1\nabla _1 \log u(x, t) \ge - \frac{1}{2} - \beta (t, n, K). \end{aligned}$$

Since the left-hand side is the smallest eigenvalue of $(Q_{ij})$, the proof is done. $\square$

## Parabolic frequency monotonicity

### Parabolic frequency

Let $(M^n,g(t))$, $t \in [0,T]$, be a complete solution to the Ricci flow. Let *u* be a solution to the (backward) conjugate heat equation $(\partial _t+\Delta )u=Ru$. Let $G(x,x_0,t)$ be the heat kernel of the heat equation (1.8) with the pole at $(x_0, 0)$. We defined in the Introduction section that

5.1 $$\begin{aligned} I(t):= & {} \int _M u^2(x,t) G(x,x_0, t) dg(t)(x), \end{aligned}$$

5.2 $$\begin{aligned} D(t):= & {} \int _M |\nabla u(x,t)|^2 G(x, x_0,t) dg(t)(x), \end{aligned}$$

5.3 $$\begin{aligned} S(t):= & {} \int _M u^2(x,t)R(x,t) G(x, x_0,t) dg(t)(x) . \end{aligned}$$

Here $dg(t)(x):=d\mu _{g(t)}$ is the Riemannian measure induced by *g*(*t*).

Below we will simply write $I(t)=\int _M u^2G dg$ and similar notations for other integrals if no confusion arises.

#### Lemma 5.1

*I*(*t*) defined in (5.1) satisfies

5.4 $$\begin{aligned} I'(t) = 2D(t)+S(t). \end{aligned}$$

#### Proof

Under the Ricci flow, the measure *d* *g*(*t*) evolves by $\partial _t (d{g(t)})=-R d {g(t)}.$ A straightforward computation shows that

$$\begin{aligned} I'(t)= & {} \int _M 2uu_t G dg +\int _M u^2 G_t dg-\int _M u^2 R G dg\\= & {} \int _M 2u(Ru-\Delta u )G dg +\int _M u^2 \Delta G dg-\int _M u^2 R G dg\\= & {} \int _M u^2 RG dg-\int _M (\Delta u^2- 2|\nabla u|^2) G dg+ \int _M \Delta u^2 G dg\\= & {} 2\int _M |\nabla u|^2 G dg+\int _M u^2 RG dg\\= & {} 2D(t)+S(t). \end{aligned}$$

$$\square $$

#### Lemma 5.2

*D*(*t*) defined in (5.2) satisfies

5.5 $$\begin{aligned} D'(t)= & {} 2 \int _M (\text {Ric}-\nabla ^2 f) (\nabla u, \nabla u) G dg+2 \int _M (\Delta _f u)^2 G dg \nonumber \\ {}{} & {} -2\int _M Ru(\Delta _f u) G dg -\int _M |\nabla u|^2 R G dg, \end{aligned}$$

where $$f=-\log G$$ and $$\Delta _f := \Delta -\langle \nabla f, \nabla \cdot \rangle $$.

#### Proof

Noticing $\partial _t|\nabla u|^2 =2\text {Ric}(\nabla u, \nabla u) +2 \langle \nabla u, \nabla u_t\rangle$ and $\partial _t dg(t)=-Rdg(t)$, we get by differentiating under the integral that

5.6 $$\begin{aligned} D'(t)= & {} 2\int _M \text {Ric}(\nabla u, \nabla u) Gdg +2\int _M \langle \nabla u, \nabla u_t \rangle G dg \nonumber \\{} & {} +\int _M |\nabla u|^2 G_t dg-\int _M |\nabla u|^2 R G dg \nonumber \\= & {} 2\int _M \text {Ric}(\nabla u, \nabla u) G dg +2\int _M \langle \nabla u, \nabla (Ru -\Delta u) \rangle G dg \nonumber \\{} & {} +\int _M |\nabla u|^2 \Delta G dg-\int _M |\nabla u|^2 R G dg \nonumber \\= & {} 2\int _M \text {Ric}(\nabla u, \nabla u) G dg+2\int _M \langle \nabla u, \nabla (Ru) \rangle G dg \nonumber \\{} & {} -2\int _M \langle \nabla u, \nabla (\Delta u) \rangle G dg +\int _M \Delta |\nabla u|^2 G dg \nonumber \\{} & {} -\int _M |\nabla u|^2 R Gdg. \end{aligned}$$

In view of the integration by parts

$$\begin{aligned} \int _M \langle \nabla u, \nabla (Ru) \rangle G dg= -\int _M Ru \Delta _f u Gdg \end{aligned}$$

and the Bochner formula

$$\begin{aligned} \Delta |\nabla u|^2 =2|\nabla ^2 u|^2 +2\langle \nabla u, \nabla \Delta u \rangle +2\text {Ric}(\nabla u, \nabla u), \end{aligned}$$

we derive from (5.6) that

5.7 $$\begin{aligned} D'(t)= & {} 4 \int _M \text {Ric}(\nabla u, \nabla u) G dg + 2\int _M |\nabla ^2 u|^2 G dg \nonumber \\{} & {} -2\int _M Ru \Delta _f u G dg-\int _M |\nabla u|^2 R G dg, \end{aligned}$$

The weighted Bochner formula

$$\begin{aligned} \Delta _f |\nabla u|^2 =2|\nabla ^2 u|^2 +2\langle \nabla u, \nabla \Delta _f u \rangle +2\text {Ric}(\nabla u, \nabla u) +2\nabla ^2 f(\nabla u, \nabla u), \end{aligned}$$

implies that

$$\begin{aligned}{} & {} 2\int _M \text {Ric}(\nabla u, \nabla u)G dg +2\int _M |\nabla ^2 u|^2 G dg\\{} & {} \quad = -2 \int _M \langle \nabla u, \nabla \Delta _f u \rangle G dg-2 \int _M \nabla ^2 f(\nabla u, \nabla u) Gdg\\{} & {} \quad = 2 \int _M (\Delta _f u)^2 G dg-2 \int _M \nabla ^2 f(\nabla u, \nabla u) G dg. \end{aligned}$$

Substituting the above identity into (5.7) produces

$$\begin{aligned} D'(t)= & {} 2 \int _M (\text {Ric}-\nabla ^2 f) (\nabla u, \nabla u) Gdg +2 \int _M (\Delta _f u)^2 G dg \\{} & {} -2\int _M Ru(\Delta _f u) G dg -\int _M |\nabla u|^2 R G dg. \end{aligned}$$

This proves (5.5). $$\square $$

#### Lemma 5.3

*S*(*t*) defined in (5.3) satisfies

5.8 $$\begin{aligned} S'(t)= & {} \int _M u^2 R^2 G dg +2\int _M u^2 |\text {Ric}|^2 G dg+2\int _M |\nabla u|^2 RG dg \nonumber \\ {}{} & {} +2\int _M u^2 R\Delta G dg +4\int _M uR\langle \nabla u, \nabla G \rangle dg. \end{aligned}$$

#### Proof

We compute using $\partial _t R =\Delta R+2|\text {Ric}|^2$ that

$$\begin{aligned} S'(t)= & {} \int _M 2uu_tRG dg+\int u^2 R_t G dg\\{} & {} +\int _M u^2 R G_t dg -\int u^2R^2 G dg\\= & {} \int _M 2u(Ru-\Delta u)RG dg+\int u^2(\Delta R+2|\text {Ric}|^2)G dg\\{} & {} +\int _M u^2 R\Delta G dg-\int u^2R^2 G dg\\= & {} \int _M u^2 R^2 G dg- 2\int _M u\Delta u RG dg +\int u^2 \Delta R Gdg \\{} & {} +2 \int _M u^2 |\text {Ric}|^2 G dg+\int _M u^2 R\Delta G dg. \end{aligned}$$

Observing

$$\begin{aligned} \int _M u^2 \Delta R G dg= & {} \int _M R \Delta (u^2 G) dg\\= & {} \int _M R\left( \Delta u^2 G +u^2 \Delta G +2\langle \nabla u^2, \nabla G \rangle \right) dg \\= & {} 2\int _M u\Delta u RG dg+2\int _M |\nabla u|^2 RG dg\\{} & {} +\int _M u^2R \Delta G dg+ 4\int _M uR\langle \nabla u, \nabla G \rangle dg, \end{aligned}$$

we deduce that

$$\begin{aligned} S'(t)= & {} \int _M u^2 R^2 G dg+2\int _M u^2 |\text {Ric}|^2 G +2\int _M |\nabla u|^2 RG dg\\{} & {} +2\int _M u^2 R\Delta G dg+ 4\int _M uR\langle \nabla u, \nabla G \rangle dg. \end{aligned}$$

$$\square $$

#### Lemma 5.4

For *I*(*t*) defined in (5.1), we have

5.9 $$\begin{aligned} I''(t)= & {} 4 \int _M (\text {Ric}-\nabla ^2 f)(\nabla u, \nabla u) G dg \nonumber \\ {}{} & {} +\int _M \left( 2\Delta _f u -Ru \right) ^2 G dg+2\int _M u^2 |\text {Ric}|^2 G dg \nonumber \\ {}{} & {} +2\int _M u^2 R\Delta G dg+4\int _M Ru\langle \nabla u, \nabla G \rangle dg. \end{aligned}$$

#### Proof

By (5.4), we have $I'(t)=2D(t)+S(t)$. Using (5.5) and (5.8), we calculate

$$\begin{aligned} I''(t)= & {} 2D'(t) +S'(t) \\= & {} 4 \int _M (\text {Ric}-\nabla ^2 f)(\nabla u, \nabla u) G dg+4 \int _M (\Delta _f u)^2 G dg \\{} & {} -4\int _M Ru(\Delta _f u) G dg -2\int _M |\nabla u|^2 R G dg \\{} & {} + \int _M u^2 R^2 G dg+2\int _M u^2 |\text {Ric}|^2 G +2\int _M |\nabla u|^2 RG dg\\{} & {} +2 \int _M u^2 R\Delta Gdg +4\int _M Ru\langle \nabla u, \nabla G \rangle dg \\= & {} 4 \int _M (\text {Ric}-\nabla ^2 f)(\nabla u, \nabla u) G dg +\int _M \left( 2\Delta _f u -Ru \right) ^2 G dg\\{} & {} +2\int _M u^2 |\text {Ric}|^2 G dg +2\int _M u^2 R\Delta G dg+4\int _M Ru\langle \nabla u, \nabla G \rangle dg. \end{aligned}$$

$$\square $$

### The nonnegative sectional curvature case

We prove Theorem 1.7 and Corollary 1.8 in this subsection.

#### Proof of Theorem 1.7

We need to estimate $I''(t)$ in (5.9) from below. Using Theorem 1.1 and $\text {Ric}\ge 0$, we get

5.10 $$\begin{aligned} \int _M (\text {Ric}+\nabla ^2 \log G)(\nabla u, \nabla u) G dg \ge -\frac{\kappa }{1-e^{-2\kappa t}} D(t). \end{aligned}$$

By Corollary 1.2, we have

$$\begin{aligned} \Delta G \ge |\nabla G|^2G^{-1} -\frac{n\kappa }{1-e^{-2\kappa t}} G. \end{aligned}$$

Since $R\ge 0$, we obtain

5.11 $$\begin{aligned} \int _M u^2 R \Delta G \ge \int _M u^2 R |\nabla G|^2G^{-1}dg -\frac{n\kappa }{1-e^{-2\kappa t}}S(t). \end{aligned}$$

Noticing $0 \le R\le n\kappa$, we estimate that

5.12 $$\begin{aligned}{} & {} 2 \int _M Ru\langle \nabla u, \nabla G \rangle dg \nonumber \\ {}\ge & {} -\int _M Ru^2 |\nabla G|^2G^{-1} dg -\int _M R |\nabla u|^2 G dg \nonumber \\\ge & {} -\int _M Ru^2 |\nabla G|^2G^{-1} dg -n\kappa D(t) \end{aligned}$$

Inserting the estimates (5.10), (5.11), and (5.12) into (5.9) yields

5.13 $$\begin{aligned} I''(t) \ge \int _M \left( 2\Delta _f u -Ru \right) ^2 G dg-\left( \frac{2 \kappa }{1-e^{-2\kappa t}} +n\kappa \right) I'(t) \end{aligned}$$

where we have used $S(t)\ge 0$ and (5.4).

Using (5.13) and the Cauchy-Schwarz inequality,

$$\begin{aligned} (I'(t))^2= & {} \left( \int _M u (2\Delta _f u-Ru)G dg \right) ^2 \\\le & {} \int _M u^2G dg \cdot \int _M \left( 2\Delta _f u -Ru \right) ^2 G dg, \end{aligned}$$

we obtain that for $F(t):=(\log I(t))'$,

$$\begin{aligned} F'(t)= & {} I(t)^{-2} \left( I''(t)-(I'(t))^2 \right) \\\ge & {} -\left( \frac{2 \kappa }{1-e^{-2\kappa t}} +n\kappa \right) F(t). \end{aligned}$$

Therefore, the quantity

$$\begin{aligned} e^{(n+2)\kappa t}(1-e^{-2\kappa t})F(t) \end{aligned}$$

is monotone nondecreasing. $\square$

Next, we prove the unique continuation property.

#### Proof of Corollary 1.8

The key point to achieve unique continuation is that the correction factor in (1.19) is asymptotic to *t* as $t\rightarrow 0$.

Suppose a solution $u=u(x,t)$ of the conjugate heat equation in $M \times [0, T)$ vanishes of infinite order at $(x_0, t_0) \in M\times (0,T)$. Since the quantity $e^{(n+2)\kappa t}(1-e^{-2\kappa t})F(t)$ is monotone nondecreasing on [0, *T*], we have for all $t\in (0,T)$,

$$\begin{aligned} F(t) \le F(T) e^{(n+2)\kappa (T-t)}\frac{1-e^{-2\kappa T}}{1-e^{-2\kappa t}} \le \frac{C}{t}, \end{aligned}$$

where

$$\begin{aligned} C:=F(T)e^{(n+2)\kappa T}(1-e^{-2\kappa T})\left( \frac{1}{2\kappa } +T \right) . \end{aligned}$$

Using $F(t)=(\log I(t))'$, we then obtain

$$\begin{aligned} I(t-t_0) \ge \frac{I(T)}{T^C}(t-t_0)^C \end{aligned}$$

for all $t\in (t_0,T)$. Noticing by Lemma 5.1 and $R\ge 0$ that $I'(t)=2D(t)+S(t)\ge 0$, we conclude that

5.14 $$\begin{aligned} I(t)\ge I(t-t_0) \ge \frac{I(T)}{T^C}(t-t_0)^C \end{aligned}$$

for all $$t\in (t_0,T)$$.

The rest of the proof is standard since the heat kernel *G* has Gaussian upper bound and the distance $d(x, x_0, t)$ are comparable in short time due to our assumption. This Gaussian bound and the assumption on the infinite vanishing order of *u* at $(x_0, t_0)$ implies, for all small $t>0$,

$$\begin{aligned} I(t) = \int _M u^2(x, t) G(x, x_0, t)dg(t)(x) \le C_N |t-t_0|^N \end{aligned}$$

for any positive integer *N*, which is a contradiction to (5.14) unless $u \equiv 0$. $\square$

### The general case

Since our assumption implies that $| \text {Ric}| \le c_n K$, taking $\alpha =2$ and $\rho =\infty$ in [2, Theorem 2.7], we have the following Li-Yau bound

5.15 $$\begin{aligned} \frac{|\nabla G|^2}{G^2} - 2 \frac{G_t}{G} \le c(n) \left( \frac{1}{t} + K \right) , \end{aligned}$$

where *c*(*n*) is a dimensional constant. Then

5.16 $$\begin{aligned} \Delta G =G_t \ge \frac{|\nabla G|^2}{2 G} - \left( \frac{c(n)}{t} +K \right) G. \end{aligned}$$

Note that this also follows from the matrix Harnack inequality in Sect. 4 after taking the trace.

Let $s_0 = \inf _{x \in M^n} R(x, 0)$. It is well known that $R(x, t) \ge s_0$ for all $t\in [0,T]$. Since $R-s_0 \ge 0$ and

5.17 $$\begin{aligned}{} & {} \int _M u^2 R\Delta G dg + 2\int _M R u\langle \nabla u, \nabla G \rangle dg \nonumber \\{} & {} \quad =\int _M u^2 (R-s_0) \Delta G dg + \int _M u^2 s_0 \Delta G dg+ 2\int _M R u\langle \nabla u, \nabla G \rangle dg \nonumber \\{} & {} \quad =\int _M u^2 (R-s_0) \Delta G dg + 2\int _M (R - s_0) u\langle \nabla u, \nabla G \rangle dg, \end{aligned}$$

we can apply (5.16) on the right-hand hand side of (5.17) to reach

$$\begin{aligned}{} & {} \int _M u^2 R\Delta G dg + 2\int _M R u\langle \nabla u, \nabla G \rangle dg\\{} & {} \quad \ge \int _M u^2 (R-s_0) \left[ \frac{|\nabla G|^2}{2 G} - \left( \frac{c(n)}{t} +K \right) G \right] dg \\{} & {} \qquad + 2\int _M (R - s_0) u\langle \nabla u, \nabla G \rangle dg. \end{aligned}$$

Using Cauchy-Schwarz inequality, we have

$$\begin{aligned}{} & {} 2 \int _M (R-s_0) u\langle \nabla u, \nabla G \rangle dg \\{} & {} \quad \ge - \frac{1}{2} \int (R-s_0) u^2 |\nabla \log G|^2 G dg - 2 \int _M (R-s_0) |\nabla u|^2 G dg. \end{aligned}$$

Hence, we obtain the estimate

$$\begin{aligned}{} & {} \int _M R u^2 \Delta G dg + 2\int _M R u\langle \nabla u, \nabla G \rangle dg \\{} & {} \quad \ge -\left( \frac{c(n)}{t} +K \right) \int _M (R-s_0) u^2 G dg \\{} & {} \qquad - 2 \int _M (R-s_0) |\nabla u|^2 G dg. \end{aligned}$$

Using this inequality, the matrix Harnack inequality in Theorem 1.3,

5.18 $$\begin{aligned} - \nabla ^2 f \ge - c_1(t) g_{ij}, \quad c_1(t) := \frac{1}{2 t} + \frac{1}{t} \beta (t, n, K) \end{aligned}$$

we deduce, from Lemma 5.4, that

$$\begin{aligned} I''(t)\ge & {} \int _M \left( 2\Delta _fu -Ru\right) ^2 G dg+2\int u^2 |\text {Ric}|^2 G dg\\{} & {} -4(c_1(t) + c(n) K) \int _M |\nabla u|^2 G dg -4 \int _M (R-s_0) |\nabla u|^2 G dg \\{} & {} - 2 \left( \frac{c(n)}{t} +K \right) \int _M (R-s_0) u^2 G dg. \end{aligned}$$

Here we have used $\text {Ric}(\nabla u, \nabla u) \ge -c(n) K |\nabla u |^2$. Using the fact that $R-s_0 \le c(n) K$ and adjusting the dimensional constant *c*(*n*), we deduce

$$\begin{aligned} I''(t)\ge & {} \int _M \left( 2\Delta _fu -Ru\right) ^2 G dg+2\int u^2 |\text {Ric}|^2 G dg\\{} & {} -2(c_1(t) + c(n) K) \left[ \int _M 2|\nabla u|^2 G dg + \int _M R u^2 G dg \right] \\{} & {} + (c_1(t) + c(n) K) \int _M R u^2 G dg \\{} & {} - 2 \left( \frac{c(n)}{t} +K \right) \int _M (R-s_0) u^2 G dg. \end{aligned}$$

Therefore

$$\begin{aligned} I''(t)\ge & {} \int _M \left( 2\Delta _fu -Ru\right) ^2 G dg+2\int u^2 |\text {Ric}|^2 G dg\\{} & {} -2(c_1(t) + c(n) K) \left[ \int _M 2|\nabla u|^2 G dg + \int _M R u^2 G dg \right] \\{} & {} - c_2(t) \int _M u^2 G dg, \end{aligned}$$

where

5.19 $$\begin{aligned} c_2(t)= c(n) K \left[ 2 (c_1(t) + c(n) K) + 2 \left( \frac{c(n)}{t} +K \right) \right] . \end{aligned}$$

This implies that

$$\begin{aligned}{} & {} I^2(t)(\log I(t))'' \\{} & {} \quad = I''(t) I(t) -(I'(t))^2 \\{} & {} \quad \ge - 2 (c_1(t) + c(n) K) I(t) \left( 2 \int _M |\nabla u|^2 G dg + \int _M Ru^2G dg \right) \\{} & {} \qquad - c_2(t) I^2(t)+ I(t) \int _M \left( 2\Delta _fu -Ru\right) ^2 G dg-(2 D(t) + S(t))^2. \end{aligned}$$

Using integration by parts, we see that

$$\begin{aligned} 2D(t) + S(t)= \int _M (-2 \Delta _f u + R u) u G dg. \end{aligned}$$

Therefore the difference of the last two terms in the preceding inequality is non-negative by Cauchy-Schwarz inequality, giving us:

5.20 $$\begin{aligned} I^2(t)(\log I(t))'' \ge - 2 (c_1(t) + c(n) K) I(t) \left[ 2 D(t) + S(t) \right] - c_2(t) I^2(t). \end{aligned}$$

Hence

5.21 $$\begin{aligned} (\log I(t))'' \ge - 2 (c_1(t) + c(n) K) \frac{ 2 D(t) + S(t)}{I(t)} - c_2(t). \end{aligned}$$

Let us recall from (5.18) and Theorem 1.3,

5.22 $$\begin{aligned} c_1(t):= & {} \frac{1}{2 t} + \frac{1}{t} \beta (t, n, K) \nonumber \\= & {} \frac{1}{2 t} + \frac{1}{t} \left[ 4 \sqrt{n K t} +C_2 (K+1)t +C_1 \sqrt{K}\, {\text {diam}}\right] \end{aligned}$$

and from (5.19)

$$\begin{aligned} c_2(t)= c(n) K \left[ 2 (c_1(t) + c(n) K) + 2 \left( \frac{c(n)}{t} +K \right) \right] . \end{aligned}$$

Let

$$\begin{aligned} Z_0 = \sup _{t \in (0, T]} [t c_2(t)]. \end{aligned}$$

Inequality (5.21) infers for $F(t):=(\log I(t))'= \frac{ 2 D(t) + S(t)}{I(t)}$,

5.23 $$\begin{aligned} F'(t) \ge -2(c_1(t) + c(n) K) F(t) - \frac{Z_0}{t}. \end{aligned}$$

Let

5.24 $$\begin{aligned} p = p(n, K, v_0, T, diam)= \sup _{t \in (0, T]} [t 2 (c_1(t) + c(n) K)] \end{aligned}$$

From the preceding inequality, we see that

$$\begin{aligned} t F'(t) \ge -p F(t) - Z_0. \end{aligned}$$

Therefore

$$\begin{aligned} \left( t^p F(t) + \frac{Z_0}{p} t^p \right) ' \ge 0 \end{aligned}$$

Thus, we have proved Theorem 1.9.

### The unweighted case

For a solution *u*(*x*, *t*) to the heat equation on ${\mathbb {R}}^n$, the monotonicity of the unweighted frequency

$$\begin{aligned} \frac{\int _{{\mathbb {R}}^n} |\nabla u(x,t)|^2 dx}{\int _{{\mathbb {R}}^n} u^2(x,t) dx} \end{aligned}$$

is equivalent to the log convexity of the energy $\int u^2 dx$. This is a classical result that can be used to prove the uniqueness of the backward heat equation (see for instance [34]). Here we extend this result to the conjugate heat equation coupled with the Ricci flow. Compared with the weighted case in this section, the curvature assumption is $\text {Ric}\ge 0$ and no upper bound of any curvature is needed. The unweighted monotonicity, however, is not strong enough to prove the unique continuation property.

#### Theorem 5.1

Let $(M^n,g(t))$, $t\in [0,T]$, be a compact Ricci flow. Let *u* be a solution to the backward conjugate heat equation (1.7). Define

$$\begin{aligned} I(t) = \int u^2(x,t) d\mu _{g(t)}. \end{aligned}$$

If $(M^n,g(t))$ has nonnegative Ricci curvature, then

$$\begin{aligned} (\log I(t))'' \ge 0. \end{aligned}$$

#### Proof

As before, we also define

$$\begin{aligned} D(t)= & {} \int |\nabla u(x,t)|^2 d\mu _{g(t)}, \\ S(t)= & {} \int u^2(x,t) R(x,t) d\mu _{g(t)}, \end{aligned}$$

and write $I(t)=\int _M u^2 dg$ for short and similar notations for other integrals.

By direct computation as for the weighted case, we have

$$\begin{aligned} I'(t)= & {} 2 \int |\nabla u|^2dg +\int u^2 R dg=2D(t)+S(t), \\ D'(t)= & {} 2\int \langle \nabla u, \nabla u_t \rangle dg +2\int \text {Ric}(\nabla u, \nabla u)dg - \int |\nabla u|^2 R dg \\= & {} -2\int u_t \Delta u dg+2\int \text {Ric}(\nabla u, \nabla u)dg - \int |\nabla u|^2 R dg, \end{aligned}$$

and

$$\begin{aligned} S'(t)= & {} 2 \int uu_t R dg+\int u^2 (\Delta R +2|\text {Ric}|^2) dg-\int u^2 R^2 dg \\= & {} 2 \int uu_t R dg+\int R(2u\Delta u +2|\nabla u|^2) \\{} & {} +2\int u^2|\text {Ric}|^2 dg-\int u^2 R^2 dg.\\ \end{aligned}$$

Using $\Delta u=Ru-u_t$, we deduce

$$\begin{aligned} I''(t)= & {} 2D'(t)+S'(t) \\= & {} -4\int u_t \Delta u dg+4\int \text {Ric}(\nabla u, \nabla u) dg \\{} & {} +2\int Ru \Delta u dg +2 \int uu_t R +2\int u^2|\text {Ric}|^2 dg -\int u^2 R^2 dg\\= & {} 4\int u_t^2 dg-4\int uu_t R dg+\int u^2R^2 dg \\{} & {} +4\int \text {Ric}(\nabla u, \nabla u) dg+2\int u^2|\text {Ric}|^2 dg \\= & {} \int (2u_t-uR)^2 dg +4\int \text {Ric}(\nabla u, \nabla u) dg+2\int u^2|\text {Ric}|^2 dg. \end{aligned}$$

Using $I'(t)=\int u(2u_t-uR)$, we then get

$$\begin{aligned}{} & {} I^2 (\log I)'' \\{} & {} \quad = \int u^2 dg\cdot \int (2u_t -uR)^2 dg- \left( \int u(2u_t-uR) dg\right) ^2 \\{} & {} \qquad +\int u^2 dg\left( 4\int \text {Ric}(\nabla u, \nabla u) dg +2\int u^2|\text {Ric}|^2 dg\right) \end{aligned}$$

The first line on the right-hand side of the above equation is nonnegative by the Cauchy-Schwarz inequality and the second line is nonnegative since $\text {Ric}\ge 0$. Therefore, we have proved the log convexity of the energy *I*(*t*). $\square$

## Matrix Harnack for the conjugate heat equation

In this section, we prove Theorem 1.5. Let’s first recall the Harnack estimate for the Ricci flow since it will be used in the proof.

### Proposition 6.1

Let $(M^n,g(t))$, $t\in (0,T)$, be a complete solution to the Ricci flow with bounded nonnegative complex sectional curvature. Define

6.1 $$\begin{aligned} M_{ij} := \Delta R_{ij} -\tfrac{1}{2}\nabla _i\nabla _j R +2R_{ikjl}R_{kl} -R_{ik}R_{jk} +\tfrac{1}{2t}R_{ij}, \end{aligned}$$

and

6.2 $$\begin{aligned} P_{kij} := \nabla _k R_{ij} -\nabla _i R_{kj}. \end{aligned}$$

Then we have

6.3 $$\begin{aligned} M(w,w)+2P(v,w,w)+{\text {Rm}}(v,w,v,w) \ge 0 \end{aligned}$$

for all $(x,t) \in M \times (0,T)$ and all vectors $v,w \in T_xM$.

### Proof

The Harnack estimate for the Ricci flow was originally proved by Hamilton [29] under the nonnegative curvature operator condition. This version stated here is a generalization due to Brendle [5]. Notice that *M* has nonnegative complex sectional curvature if and only $M\times {\mathbb {R}}^2$ has nonnegative isotropic curvature, which is an observation of Ni and Wolfson [59]. $\square$

The next step is to derive an evolution inequality for

6.4 $$\begin{aligned} Z_{ij}:=R_{ij} -\nabla _i\nabla _j \log u -\eta (t) g_{ij}. \end{aligned}$$

### Proposition 6.2

Let $(M^n,g(t))$, *u*, and $\eta (t)$ be the same as in Theorem 1.5. Then $Z_{ij}$ defined in (6.4) satisfies

6.5 $$\begin{aligned} \tfrac{1}{2}(\partial _t+\Delta )Z_{ij} \ge Z^2_{ij}-R_{ikjl}Z_{kl}-\tfrac{1}{2}R_{ik}Z_{jk}-\tfrac{1}{2}R_{jk}Z_{ik}+2\eta Z_{ij} -\nabla _k Z_{ij} \nabla _k v. \end{aligned}$$

### Proof

For simplicity, we write $v=\log u$ and

$$\begin{aligned} H_{ij} = \nabla _i \nabla _j \log u. \end{aligned}$$

Then the calculations in Sect. 2 with $\varepsilon =-1$ and $\delta =1$ apply to this setting and we obtain from (2.2) that

6.6 $$\begin{aligned} (\partial _t+\Delta )H_{ij}= & {} \nabla _i\nabla _j R - (2R_{ikjl}H_{kl}-R_{ik}H_{jk}-R_{jk}H_{ik}) \nonumber \\{} & {} - 2 \left( H^2_{ij} +R_{ikjl}\nabla _k v \nabla _l v +\nabla _k H_{ij} \nabla _k v \right) \nonumber \\{} & {} +2 (\nabla _iR_{jk}+\nabla _jR_{ik}-\nabla _kR_{ij})\nabla _k v. \end{aligned}$$

Under the Ricci flow, we have (see [16, page 112])

$$\begin{aligned} (\partial _t - \Delta ) R_{ij} =2R_{ikjl}R_{kl}-2R_{ik}R_{jk}. \end{aligned}$$

Hence,

6.7 $$\begin{aligned} (\partial _t + \Delta ) R_{ij} =2\Delta R_{ij} + 2R_{ikjl}R_{kl}-2R_{ik}R_{jk}. \end{aligned}$$

We also notice that

6.8 $$\begin{aligned} (\partial _t + \Delta ) (\eta (t)g_{ij}) = \eta '(t)g_{ij} -2\eta (t)R_{ij}. \end{aligned}$$

Combining (6.6), (6.7), and (6.8) together, we obtain that

$$\begin{aligned}{} & {} \tfrac{1}{2}(\partial _t+\Delta )Z_{ij} \\{} & {} \quad = \tfrac{1}{2} (\partial _t+\Delta )R_{ij}-\tfrac{1}{2}(\partial _t+\Delta )H_{ij}-\tfrac{1}{2}(\partial _t+\Delta )(\eta (t)g_{ij}) \\{} & {} \quad = \Delta R_{ij} +R_{ikjl}R_{kl}-R_{ik}R_{jk} -\tfrac{1}{2}\nabla _i\nabla _j R + H^2_{ij} \\{} & {} \qquad +R_{ikjl}H_{kl}-\tfrac{1}{2}R_{ik}H_{jk}-\tfrac{1}{2}R_{jk}H_{ik} +R_{ikjl}\nabla _k v \nabla _l v \\{} & {} \qquad -\nabla _k (Z_{ij}-R_{ij})\nabla _k v - (\nabla _iR_{jk}+\nabla _jR_{ik}-\nabla _kR_{ij})\nabla _k v \\{} & {} \qquad -\tfrac{\eta '(t)}{2}g_{ij} +\eta (t)R_{ij} \\{} & {} \quad = \Delta R_{ij} -\tfrac{1}{2} \nabla _i \nabla _j R +2R_{ikjl}R_{kl}-R_{ik}R_{jk} +R_{ikjl}\nabla _k v \nabla _l v \\{} & {} \qquad + H^2_{ij} -R_{ikjl}R_{kl} + R_{ikjl}H_{kl}-\tfrac{1}{2}R_{ik}H_{jk}-\tfrac{1}{2}R_{jk}H_{ik}\\{} & {} \qquad -\nabla _k Z_{ij}\nabla _k v - (\nabla _iR_{jk}+\nabla _jR_{ik}-2\nabla _kR_{ij})\nabla _k v \\{} & {} \qquad -\tfrac{\eta '(t)}{2}g_{ij} +\eta (t)R_{ij}. \end{aligned}$$

Using (6.1), (6.2), and

$$\begin{aligned}{} & {} -(\nabla _iR_{jk}+\nabla _jR_{ik}-2\nabla _kR_{ij})\nabla _k v \\{} & {} \quad = (\nabla _k R_{ij} -\nabla _i R_{jk})\nabla _k v +(\nabla _k R_{ij} -\nabla _j R_{ik})\nabla _k v \\{} & {} \quad = (P_{kij}+P_{kji})\nabla _k v, \end{aligned}$$

we get

6.9 $$\begin{aligned}{} & {} \tfrac{1}{2}(\partial _t+\Delta )Z_{ij} \nonumber \\{} & {} \quad = M_{ij}-\tfrac{1}{2t}R_{ij}+(P_{kij}+P_{kji})\nabla _k v+ R_{ikjl}\nabla _k v \nabla _l v -\nabla _k Z_{ij}\nabla _k v \nonumber \\{} & {} \qquad +H^2_{ij}-R_{ikjl}R_{kl} + R_{ikjl}H_{kl} -\tfrac{1}{2}R_{ik}H_{jk}-\tfrac{1}{2}R_{jk}H_{ik} \nonumber \\{} & {} \qquad -\tfrac{\eta '(t)}{2}g_{ij} +\eta (t)R_{ij}. \end{aligned}$$

Next, we compute using (6.4) that

6.10 $$\begin{aligned}{} & {} H^2_{ij}-R_{ikjl}R_{kl} + R_{ikjl}H_{kl} -\tfrac{1}{2}R_{ik}H_{jk}-\tfrac{1}{2}R_{jk}H_{ik} \nonumber \\{} & {} \quad = (R_{ik}-Z_{ik}-\eta g_{ik})(R_{jk}-Z_{jk}- \eta g_{jk}) -R_{ikjl}(Z_{kl}+ \eta g_{kl}) \nonumber \\{} & {} \qquad -\tfrac{1}{2}R_{ik}(R_{jk}-Z_{jk}-\eta g_{jk})-\tfrac{1}{2}R_{jk}(R_{ik}-Z_{ik}-\eta g_{ik}) \nonumber \\{} & {} \quad = Z^2_{ij}-R_{ikjl}Z_{kl}-\tfrac{1}{2}R_{ik}Z_{jk}-\tfrac{1}{2}R_{jk}Z_{ik}+2\eta Z_{ij}+\eta ^2g_{ij}-2\eta R_{ij}. \end{aligned}$$

Substituting (6.10) into (6.9) produces

6.11 $$\begin{aligned}{} & {} \tfrac{1}{2}(\partial _t+\Delta )Z_{ij} \nonumber \\{} & {} \quad = M_{ij}+(P_{kij}+P_{kji})\nabla _k v+ R_{ikjl}\nabla _k v \nabla _l v -\nabla _k Z_{ij}\nabla _k v \nonumber \\ {}{} & {} \qquad +Z^2_{ij}-R_{ikjl}Z_{kl}-\tfrac{1}{2}R_{ik}Z_{jk}-\tfrac{1}{2}R_{jk}Z_{ik}+2\eta Z_{ij}\nonumber \\{} & {} \qquad +\eta ^2g_{ij}-\eta R_{ij}-\tfrac{\eta '(t)}{2}g_{ij}-\tfrac{1}{2t}R_{ij}, \end{aligned}$$

Using (1.15) and $\text {Ric}\le \kappa g$, we have

6.12 $$\begin{aligned}{} & {} \eta ^2g_{ij}-\eta R_{ij}-\tfrac{\eta '(t)}{2}g_{ij}-\tfrac{1}{2t}R_{ij} \nonumber \\{} & {} \quad \ge \tfrac{1}{2t}(\kappa g_{ij}-R_{ij}) +\eta (\kappa g_{ij}-R_{ij}) . \end{aligned}$$

In view of (6.3) and (6.12), we conclude that

$$\begin{aligned} \tfrac{1}{2}(\partial _t+\Delta )Z_{ij} \ge Z^2_{ij}-R_{ikjl}Z_{kl}-\tfrac{1}{2}R_{ik}Z_{jk}-\tfrac{1}{2}R_{jk}Z_{ik}+2\eta Z_{ij}-\nabla _k Z_{ij}\nabla _k v. \end{aligned}$$

Hence, (6.5) is proved. $\square$

We are ready to prove Theorem 1.5.

### Proof of Theorem 1.5

By Proposition 6.2,

6.13 $$\begin{aligned} \tfrac{1}{2}(\partial _t+\Delta )Z_{ij} +\nabla _k Z_{ij} \nabla _k v \ge Z^2_{ij}-R_{ikjl}Z_{kl}-\tfrac{1}{2}R_{ik}Z_{jk}-\tfrac{1}{2}R_{jk}Z_{ik}+2\eta Z_{ij}. \end{aligned}$$

If (*M*, *g*(*t*)) is compact, it follow from $\eta (t)\rightarrow \infty$ as $t\rightarrow T$ that $Z_{ij} \le 0$ as $t \rightarrow T^-$. Noticing that each term on the right-hand side of (6.13) satisfies the null-eigenvector condition, we conclude using Hamilton’s tensor maximum principle (see [28] or [16, Theorem 3.3]) that $Z_{ij} \le 0$ on $M\times (0,T)$.

When (*M*, *g*(*t*)) is complete noncompact, one can proceed as in subsection 3.2 and use the maximum principle (see [10, Theorem 12.22] to prove the estimate. We omit the technical details here. $\square$

Next, we prove Corollary 1.6.

### Proof of Corollary 1.6

Note that the function

$$\begin{aligned} \eta _0(t)=\frac{\kappa }{1-e^{-2\kappa (T-t)}} +\sqrt{\frac{\kappa }{2t}} \end{aligned}$$

satisfies $\eta _0' \le 2\eta _0^2 -2\kappa \eta _0 -\frac{\kappa }{t}$ and $\eta _0(t)\rightarrow \infty$ as $t\rightarrow T$. Hence, the inequality (1.17) follows from choosing $\eta =\eta _0$ in (1.16). $\square$

Below we present an improvement of Theorem 1.5 when the Ricci flow is ancient.

### Theorem 6.3

Let $(M^n,g(t))$, $t\in (-\infty ,T)$, be a compact ancient solution to the Ricci flow with bounded nonnegative complex sectional curvature. Let $u: M \times [t_0, T] \rightarrow (0,\infty )$, $-\infty \le t_0<T$, be a positive solution to the backward conjugate heat equation $u_t+\Delta _{g(t)} u=Ru$. Suppose that $\text {Ric}(x,t) \le \kappa (x,t) g$ for some $\kappa >0$ and for all $(x,t)\in M\times [t_0,T]$. Then we have

$$\begin{aligned} \text {Ric}-\nabla ^2 \log u -\frac{\kappa }{1-e^{-2\kappa (T-t)}} g \le 0, \end{aligned}$$

for all $$(x,t) \in M \times (t_0,T)$$.

### Remark 6.1

In Theorem 6.3, it suffices to assume the weaker condition that $M\times {\mathbb {R}}$ has nonnegative isotropic curvature, in view of [3] and [42, Proposition 6.2].

### Proof of Theorem 6.3

The proof is almost identical to that of Theorem 1.5. The difference is that we get the improved Harnack estimate (compared with (6.3))

$$\begin{aligned} M(w,w)+2P(v,w,w)+{\text {Rm}}(v,w,v,w) \ge \tfrac{1}{2t}\text {Ric}(w,w) \end{aligned}$$

on ancient Ricci flows (see [29] or [5]). As a result, the ordinary differential inequality for $\eta (t)$ becomes

6.14 $$\begin{aligned} \eta '\le 2\eta ^2 -2\kappa \eta . \end{aligned}$$

The theorem follows from the fact that the function

$$\begin{aligned} \eta (t):=\frac{\kappa }{1-e^{-2\kappa (T-t)}} \end{aligned}$$

solves (6.14) with equality on $(t_0,T)$ and satisfies $\eta (t) \rightarrow \infty$ as $t\rightarrow T$. $\square$

We get space-time gradient estimates for $\log u$ by tracing the matrix Li–Yau–Hamilton estimates.

### Corollary 6.4

Let $(M^n,g(t))$ and *u* be the same as in Theorem 1.5. Then

$$\begin{aligned} R-\Delta \log u - \frac{n\kappa }{1-e^{-2\kappa (T-t)}} -n\sqrt{\frac{\kappa }{2t}} \le 0 \end{aligned}$$

for all $$(x,t)\in M\times (0,T)$$.

### Corollary 6.5

Let $(M^n,g(t))$ and *u* be the same as in Theorem 6.3. Then

$$\begin{aligned} R-\Delta \log u - \frac{n\kappa }{1-e^{-2\kappa (T-t)}} \le 0 \end{aligned}$$

for all $$(x,t)\in M\times (0,T)$$.

Classical-type Harnack inequalities follow from integrating the above estimates. It is an interesting question whether the above gradient estimates hold under nonnegative Ricci or sectional curvature.

As an application of Theorem 1.5, we prove Proposition 1.10.

### Proof of Proposition 1.10

By (1.17), we have $\text {Ric}-\nabla ^2 \log u \le \frac{k(t)}{2(T-t)}$ with

$$\begin{aligned} \frac{k(t)}{(T-t)}= \frac{2\kappa }{1-e^{-2\kappa (T-t)}} +\sqrt{\frac{2\kappa }{t}}. \end{aligned}$$

By the work of Baldauf and Kim [4], the correction factor is given by

$$\begin{aligned} e^{\int \frac{1-k(t)}{T-t} dt} = \frac{1}{T-t} e^{\int \left( \frac{-2\kappa }{1-e^{-2\kappa (T-t)}} -\sqrt{2\kappa } \frac{1}{\sqrt{t}} \right) dt } =\frac{1}{T-t} (e^{2\kappa (T-t)}-1) e^{-\sqrt{8\kappa t}}. \end{aligned}$$

Therefore, we have proved Proposition 1.10. $\square$

Proposition 1.10 implies a unique continuation result.

### Corollary 6.6

Let $(M^n,g(t))$, $t\in [0,T]$, be a solution to the Ricci flow with nonnegative complex sectional curvature and $\text {Ric}\le \kappa g$ for some $\kappa >0$. Suppose that a solution *u*(*x*, *t*) of the heat equation (1.8) on $M\times [0,T]$ vanishes of infinity order at $(x_0, t_0) \in M\times (0,T)$. Then $u\equiv 0$ in $M\times [0,T]$.

### Proof of Corollary 6.6

The key point is that the correction factor in (1.21) is asymptotic to $(T-t)$ as $t \rightarrow T$. The proof is similar to that of Corollary 1.8 and we omit the details. $\square$

## An improvement of Hamilton’s matrix estimate

We present the proof of Theorem 1.4 in this section.

### Proof of Theorem 1.4

We write $v=\log u$ and $H_{ij}=\nabla _i\nabla _j \log u$. By a straightforward calculation as in Section 2 or [30], we derive that

$$\begin{aligned} (\partial _t -\Delta )H_{ij}= & {} 2H^2_{ij}+2R_{ikjl}H_{kl}-R_{ik}H_{jk}-R_{jk}H_{ik}+2R_{ikjl}\nabla _k v \nabla _l v \\{} & {} +2\nabla _k H_{ij}\nabla _k v +(\nabla _l R_{ij} -\nabla _iR_{jl}-\nabla _j R_{il})\nabla _l v. \end{aligned}$$

Compared to (3.1) in the Ricci flow case, here we have the addition term $(\nabla _l R_{ij} -\nabla _iR_{jl}-\nabla _j R_{il})\nabla _l v$. As in the proof of Theorem 1.3 in Sect. 4, we consider $Q_{ij}:=tH_{ij}$, which satisfies

7.1 $$\begin{aligned} (\partial _t -\Delta )Q_{ij}= & {} \frac{1}{t}Q_{ij}+\frac{2}{t}Q^2_{ij}+2R_{ikjl}Q_{kl}-R_{ik}Q_{jk}-R_{jk}Q_{ik} \nonumber \\ {}{} & {} +2tR_{ikjl}\nabla _k v \nabla _l v +2\nabla _k Q_{ij}\nabla _k v \nonumber \\ {}{} & {} +t(\nabla _l R_{ij} -\nabla _iR_{jl}-\nabla _j R_{il})\nabla _l v. \end{aligned}$$

Let $\lambda _1$ be the smallest eigenvalue of $Q_{ij}$. Using $|\nabla \text {Ric}|\le L$, we estimate the last term

$$\begin{aligned} t(\nabla _l R_{ij} -\nabla _iR_{jl}-\nabla _j R_{il})\nabla _l v \ge -3Lt |\nabla v| g_{ij} \ge -3t(L^{\frac{2}{3}}|\nabla v|^2 +L^{\frac{4}{3}})g_{ij}. \end{aligned}$$

As in the proof of Theorem 1.3, we have the estimates

$$\begin{aligned} R_{ikjl}Q_{kl}\xi _i\xi _j\ge & {} (2n-1)K\lambda _1 -Kt\Delta v, \\ R_{ikjl}\nabla _k v \nabla _l v \xi _i\xi _j\ge & {} -K |\nabla v|^2, \end{aligned}$$

Therefore, we deduce from (7.1) that at a negative minimum point $(x_0,t_0)\in M\times [0,t_0]$ of $\lambda _1$,

7.2 $$\begin{aligned} 2\lambda _1^2\le & {} -\lambda _1-2(2n-1)Kt\lambda _1+2Kt^2\Delta v +2Kt^2|\nabla v|^2 \nonumber \\ {}{} & {} +3L^{\frac{2}{3}}t^2|\nabla v|^2 +3L^{\frac{4}{3}}t^2. \end{aligned}$$

From now on, we assume $u=G(x,t,y)$ is the heat kernel and estimate $\Delta \log u$ and $|\nabla \log u|$. First, applying Hamilton’s gradient bound [30, Theorem 1.1] to $u(x,t+t_0,y)$ yields for any $t_0>0$ that

7.3 $$\begin{aligned} (t-(t_0/2))|\nabla \log u|^2 \le (1+2(n-1)K(t-t_0/2))\log \frac{A}{u}, \end{aligned}$$

where $A=\sup \{u(x,t):(x,t)\in M\times [t_0/2,t_0]\}$. According to [72], we have

7.4 $$\begin{aligned} \log \frac{A}{u}\le & {} 2 \log C_1 +4C_2Kt_0+\frac{d^2(x,y)}{3t_0}+C_3\sqrt{K}\frac{{\text {Diam}}}{\sqrt{t_0}} \nonumber \\ {}\le & {} C_4(1+K+Kt_0)+\frac{{\text {Diam}}^2}{2t_0}, \end{aligned}$$

where $C_1$, $C_2$, $C_3$, and $C_4$ are dimensional constants. Substituting (7.4) into (7.3) produces

7.5 $$\begin{aligned} t_0|\nabla \log u|^2 \le 2(1+(n-1)Kt_0)\left( C_4(1+K+Kt_0)+\frac{{\text {Diam}}^2}{2t_0}\right) . \end{aligned}$$

Next, we apply the Laplacian estimate (see [10, Theorem E.35]) to the function $u(x,t+t_0,y)$ and get

$$\begin{aligned} (t-t_0/2)\left( \Delta \log u +2|\nabla \log u|^2 \right) \le (1+(n-1)K(t-t_0/2))\left( n+4\log \frac{A}{u}\right) . \end{aligned}$$

At $t=t_0$, we obtain using (7.4) that

7.6 $$\begin{aligned}{} & {} t_0(\Delta \log u +2|\nabla \log u|^2) \nonumber \\{} & {} \quad \le (2+(n-1)Kt_0)\left( n+4C_4(1+K+Kt_0)+ 2\frac{{\text {Diam}}^2}{t_0}\right) \end{aligned}$$

Since $t_0>0$ is arbitrary, we conclude that (7.5) and (7.6) are valid for any $t_0=t>0$. Combining (7.5) and (7.6), we estimate that

$$\begin{aligned} B:= & {} 2Kt \Delta v +2Kt |\nabla v|^2 +3L^{\frac{2}{3}}t|\nabla v|^2 \\ {}\le & {} 2K(2+(n-1)Kt_0)\left( n+4C_4(1+K+Kt_0)+ 2\frac{{\text {Diam}}^2}{t}\right) \\ {}{} & {} +3L^{\frac{2}{3}}(2(1+(n-1)Kt_0))\left( C_4(1+K+Kt_0)+\frac{{\text {Diam}}^2}{2t}\right) \\ {}\le & {} 2nK(2+(n-1)Kt) +C_5(K+L^{\frac{2}{3}})(1+Kt)(1+K+Kt) \\ {}{} & {} + \left( 4K(2+(n-1)Kt)+3L^{\frac{2}{3}}(1+(n-1)Kt) \right) \frac{{\text {Diam}}^2}{t}, \end{aligned}$$

where $C_5$ depends only on the dimension. Now, (7.2) implies,

7.7 $$\begin{aligned} 2\lambda _1^2 +\lambda _1+2(2n-1)Kt\lambda _1 \le tB +3L^{\frac{4}{3}}t^2. \end{aligned}$$

Note that if $ax^2+bx \le c$ with $a>0$, $b>0$, and $c>0$, then we have the lower bound

$$\begin{aligned} x \ge \frac{-b-\sqrt{b^2+4ac}}{2a} \ge -\frac{b+\sqrt{ac}}{a}. \end{aligned}$$

Therefore, we deduce from (7.7) that

$$\begin{aligned} \lambda _1\ge & {} -\left( \frac{1}{2}+(2n-1)Kt + \frac{1}{2}\sqrt{2(tB+3L^{\frac{4}{3}}t^2)}\right) \\\ge & {} -\left( \frac{1}{2}+(2n-1)Kt +\frac{1}{2}\sqrt{tB}+\frac{\sqrt{3}}{2}L^{\frac{2}{3}}t \right) . \end{aligned}$$

The desired estimate for $u=G(x,t,y)$ then follows by noting

$$\begin{aligned} \sqrt{tB}\le t \gamma (t,n,K,L). \end{aligned}$$

Finally, we can follow the same argument in Section 4 or [72] to show that the desired estimate holds from any positive solution to the heat equation. This completes the proof. $\square$

## Data Availability

Data sharing is not applicable to this article as no data sets were generated or analyzed during the current study.
